# Public and health professional epidemic risk perceptions in countries that are highly vulnerable to epidemics: a systematic review

**DOI:** 10.1186/s40249-021-00927-z

**Published:** 2022-01-06

**Authors:** Nada Abdelmagid, Francesco Checchi, Bayard Roberts

**Affiliations:** 1 Faculty of Public Health & Policy, London School of Hygiene & Tropical Medicine, 15-17 Tavistock Place, London, WC1H 9SH UK; 2 Faculty of Epidemiology and Population Health, London School of Hygiene & Tropical Medicine, Keppel Street, London, WC1E 7HT UK

**Keywords:** Risk perception, Epidemic, Vulnerability

## Abstract

**Background:**

Risk communication interventions during epidemics aim to modify risk perceptions to achieve rapid shifts in population health behaviours. Exposure to frequent and often concurrent epidemics may influence how the public and health professionals perceive and respond to epidemic risks. This review aimed to systematically examine the evidence on risk perceptions of epidemic-prone diseases in countries highly vulnerable to epidemics.

**Methods:**

We conducted a systematic review using PRISMA standards. We included peer-reviewed studies describing or measuring risk perceptions of epidemic-prone diseases among the general adult population or health professionals in 62 countries considered highly vulnerable to epidemics. We searched seven bibliographic databases and applied a four-stage screening and selection process, followed by quality appraisal. We conducted a narrative meta-synthesis and descriptive summary of the evidence, guided by the Social Amplification of Risk Framework.

**Results:**

Fifty-six studies were eligible for the final review. They were conducted in eighteen countries and addressed thirteen epidemic-prone diseases. Forty-five studies were quantitative, six qualitative and five used mixed methods. Forty-one studies described epidemic risk perceptions in the general public and nineteen among health professionals. Perceived severity of epidemic-prone diseases appeared high across public and health professional populations. However, perceived likelihood of acquiring disease varied from low to moderate to high among the general public, and appeared consistently high amongst health professionals. Other occupational groups with high exposure to specific diseases, such as bushmeat handlers, reported even lower perceived likelihood than the general population. Among health professionals, the safety and effectiveness of the work environment and of the broader health system response influenced perceptions. Among the general population, disease severity, familiarity and controllability of diseases were influential factors. However, the evidence on how epidemic risk perceptions are formed or modified in these populations is limited.

**Conclusions:**

The evidence affords some insights into patterns of epidemic risk perception and influencing factors, but inadequately explores what underlies perceptions and their variability, particularly among diseases, populations and over time. Approaches to defining and measuring epidemic risk perceptions are relatively underdeveloped.

**Graphical Abstract:**

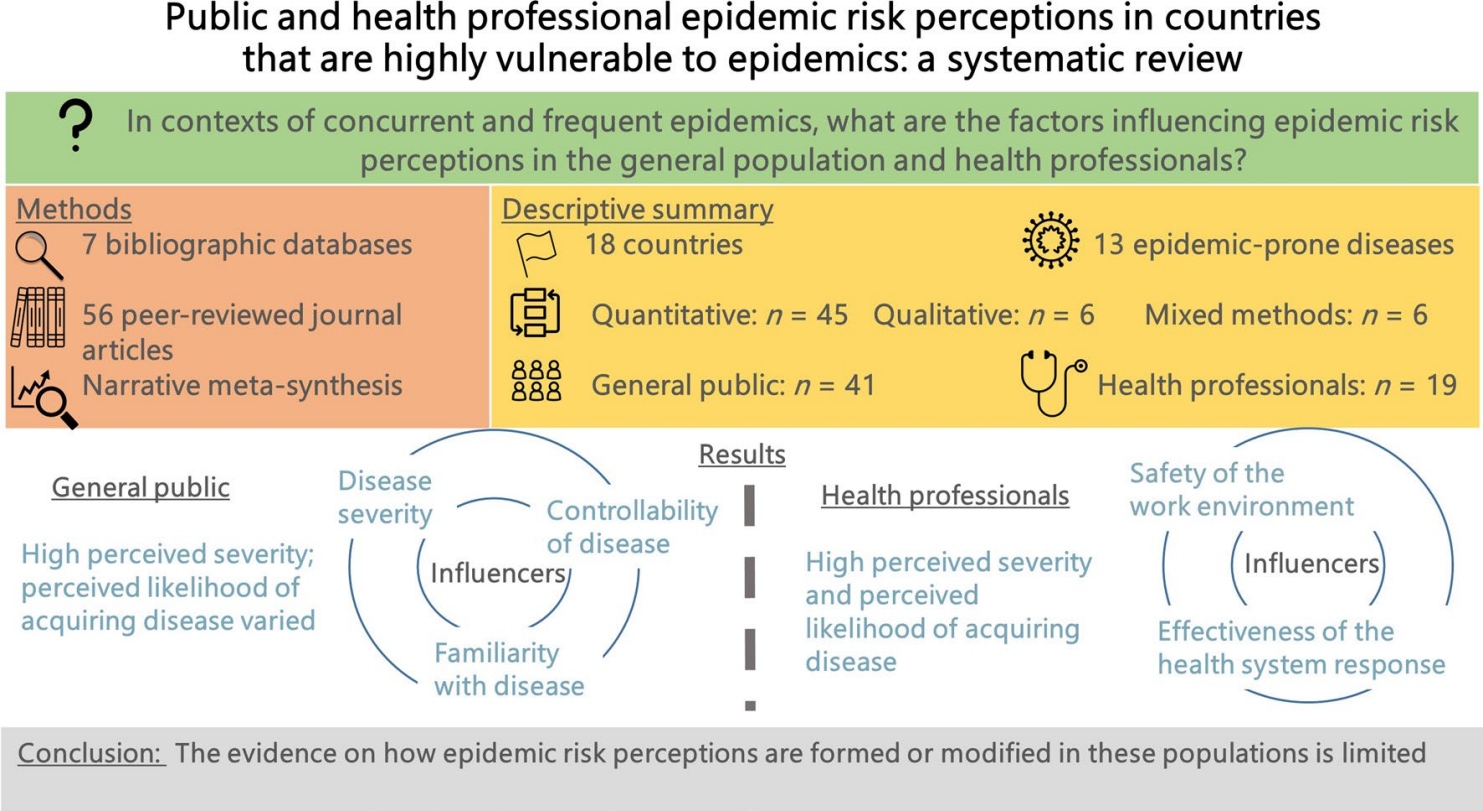

**Supplementary Information:**

The online version contains supplementary material available at 10.1186/s40249-021-00927-z.

## Background

Although the twenty-first century saw a rapid decline in global mortality attributable to infectious diseases, they continue to account for high morbidity and mortality in low-income countries [1]. Epidemics of infectious diseases may arise and propagate faster than before [2] due to increased social mixing and exposure to wild animal reservoirs and challenges with timely detection and containment [3, 4].

A 2016 analysis suggested that 22 of the 25 most epidemic-vulnerable countries are in Africa, particularly concentrated across the Sahel region, and that vulnerability correlates with recent or ongoing conflict [5]. Low-income countries are generally the least well-prepared [6], particularly in regions at elevated risk of emerging zoonotic infections [7].

An individual’s subjective judgement of a health threat—or *risk perception*—is central to key health behaviour change theories, including the Health Belief Model [8], the Protection Motivation Theory [9], the Extended Parallel Process Model [10], and the Risk Perception Attitude framework [11]. These theories generally assume that risk perceptions are an essential precursor of protective health behaviours. While this assumption has not been consistently borne out in individual studies, meta-analyses suggest a modest to moderate influence of risk perception on health behaviours [12–15].

Three theoretical approaches seek to explain risk perception. The psychometric paradigm in psychology theorizes that cognition and emotion play a role in the formation of risk perceptions, by influencing information processing and judgment for decision-making [16]. The cultural theory of risk in sociology and anthropology posits that risk is non-objective and perceptions are determined by an individual’s sociocultural reality [17]. Among multidisciplinary models, the Social Amplification of Risk Framework (SARF) ties technical assessments of risk with psychological, sociological, and cultural perspectives, modulated by social and individual factors [18].

Risk communication is a fundamental intervention in epidemic responses, and is defined by the World Health Organization as the “the real-time exchange of information, advice and opinions between experts, community leaders, or officials and the people who are at risk”, with the implicit assumption that this process will instigate appropriate individual perceptions and inform behaviour [19]. However, risk perceptions are subject to other influences [20], including individual numeracy [21], prior experiences and imminence of the threat [22].

Highly epidemic-vulnerable countries are likely to also experience insecurity, poverty and underperforming health services [5, 6]. Here, populations and responders are often confronted with concurrent and competing risks to life, against limited resources [23]. This context is likely to influence how the general population health professionals tasked with their care and protection, perceive and make decisions about health risks. Studies in low-income multi-hazard contexts indicate that environmental risk perceptions and prioritisation are influenced by hazard characteristics (e.g. chronicity) [24], individual factors (e.g. socioeconomic status) [24], and collective coping capacity [25, 26], and that risk perceptions vary within groups and over time [26].

There is insufficient evidence on the effectiveness of epidemic risk communication interventions in low and middle-income settings [27]. A thorough understanding is required of how risk perceptions of epidemics are constructed by individuals from the general population and health professionals, the factors influencing these risk perceptions and how they interact in a context of high vulnerability to epidemics. Such insight is essential for informing effective and contextualised epidemic risk communication interventions. This review aimed to examine the existing evidence on risk perceptions of epidemic-prone diseases among the public and health professionals in highly epidemic-vulnerable countries. We also examined how risk perception has been conceptualised and measured by researchers in these settings.

## Methodology

The review is designed and reported as per the PRISMA Statement [28]. The inclusion and exclusion criteria are described in Table 1.

**Table 1:** Inclusion and exclusion criteria

Category	Included	Excluded
Population of interest	• Studies of populations in one or more of 62 countries considered most vulnerable to epidemics. These have been identified as follows: a. The top 50 countries from the 2016 Infectious Disease Vulnerability Index: Somalia, Central African Republic, Chad, South Sudan, Mauritania, Angola, Haiti, Afghanistan, Niger, Madagascar, Democratic Republic of the Congo, Mali, Guinea-Bissau, Benin, The Gambia, Liberia, Guinea, São Tomé and Principe, Sierra Leone, Burkina Faso, Comoros, Yemen, Eritrea, Togo, Mozambique, Republic of the Congo (Congo Brazzaville), Nigeria, Côte d’Ivoire, Malawi, Sudan, Djibouti, Pakistan, Timor-Leste, Senegal, Zimbabwe, Papua New Guinea, Tanzania, Lesotho, Burundi, Laos, Cambodia, Rwanda, Eswatini (formerly Swaziland), Uganda, Solomon Islands, Democratic People's Republic of Korea, Ethiopia, Kenya, Kiribati and Cameroon [5] b. 12 additional countries from The World Bank’s fragile and conflict-affected states lists of 2016–2020: Kosovo, Marshall Islands, Federated States of Micronesia, Myanmar, Tuvalu, Palestine, Bosnia & Herzegovina, Iraq, Lebanon, Libya, Syria, Venezuela [104] • Population groups of interest: a. General public (aged 15 years or more) b. Health professionals (service providers, managers, planners policy makers)	Studies of populations in countries other than the 62 eligible countries Studies of nationals from one or more of the 62 countries residing in other nations (e.g. refugees and migrants)
Intervention	• Any epidemic-prone disease, defined by the WHO as an infectious disease that typically leads to outbreaks and/or epidemics [105], and that typically manifests as an acute clinical illness • Eligible studies may explore one or more epidemic-prone diseases, and may be implemented before, during or after an outbreak	• Chronic infectious diseases, namely HIV/AIDS, tuberculosis, leprosy, chronic viral hepatitis and all sexually-transmitted infections • Vaccines or other epidemic preparedness or response measures
Outcome of interest	• Measures or descriptions of risk perceptions of an epidemic-prone diseases and/or • Measures or descriptions of factors associated with risk perceptions of an epidemic-prone disease Definition of risk perceptions of an epidemic-prone diseases: • Beliefs about potential harm due to the epidemic-prone disease in question [106] • Eligible studies explored one or more of the following three dimensions of perceived risk from an epidemic-prone disease [106]: • Likelihood (the probability that one will be harmed by the epidemic-prone disease) • Susceptibility (an individual’s physical vulnerability to the epidemic-prone disease) • Severity (the extent of harm the epidemic-prone disease would cause)	Intentions to adopt or adoption of epidemic preparedness measures Intentions to adopt, adoption of or adherence to disease prevention behaviours
Study design, publication types, language and date search restrictions	Study design: all primary, observational, mixed method, quantitative and qualitative study designs Publication types: peer-reviewed articles journals for which the full text could be accessed Language: no language restrictions in the search. Data extraction limited to English results Date: Studies published since January 2011. The search period reflects recent and current vulnerability to epidemics in the countries selected	Study design: literature or systematic reviews, experimental studies Publication types: editorials, letters to the editor, commentaries, books, book chapters, conference proceedings, opinion pieces, news articles, dissertations or theses, reports, peer-reviewed articles journals for which the full text cannot be accessed

### Search strategy, study screening and selection

We searched seven bibliographic databases to cover the multiple disciplines of risk perception research: EMBASE, Global Health, MEDLINE, PsycINFO, Africa-Wide Information, CINAHL Plus and Web of Science. The search terms covered three concepts: risk perception, epidemic-prone diseases, and eligible countries. Since the concept of ‘risk’ does not translate directly into many languages spoken in the targeted countries, we included search terms for ‘risk perception’ that have been used to study similar concepts, or that hold neutral (e.g. likelihood) or positive connotations (e.g. safety). We also perused related systematic reviews to identify additional synonyms for these concepts [29–31]. Additional file 1 shows the detailed list of search terms and search strategies used. The search was not limited by language although data extraction was limited to English results. The search was restricted to citations published on or after January 2011, and was conducted on 28 December 2021.

**Fig. 1:**
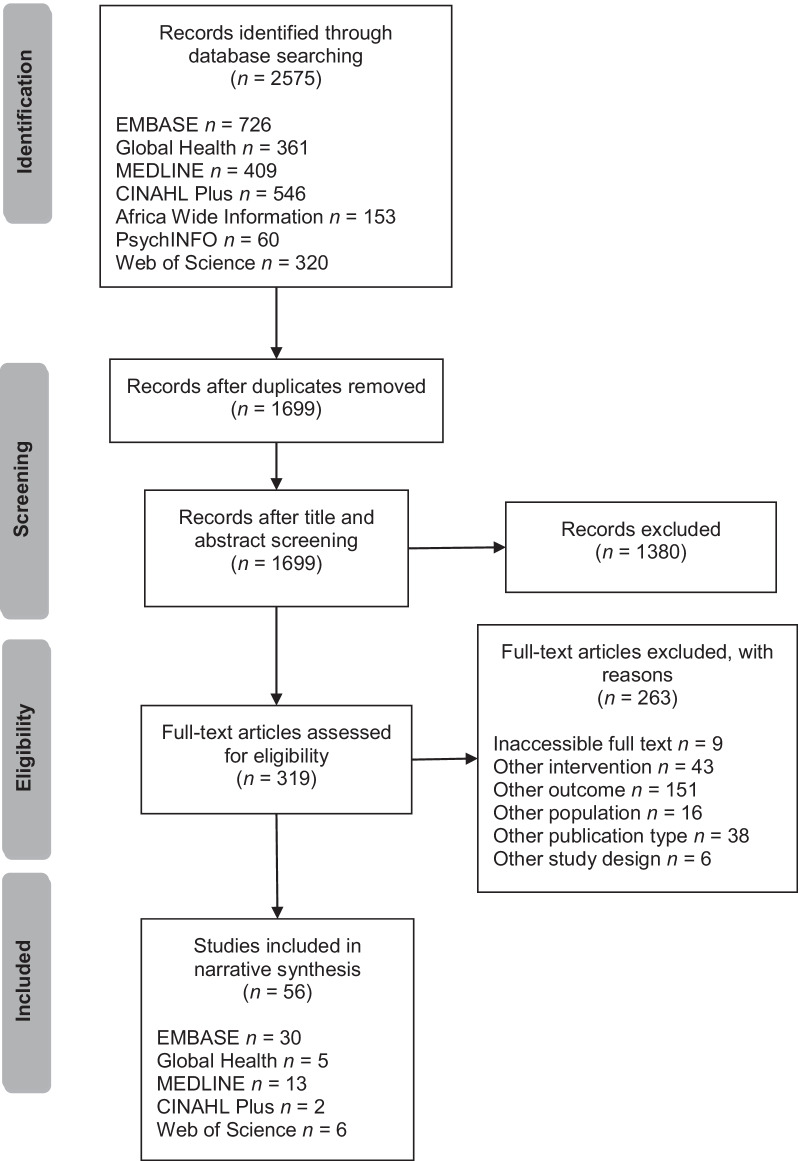
Results of study screening and selection process

We exported all citations into EndNote (Version X9, Clarivate Analytics, Philadelphia, United States of America) for screening and selection. This phase was carried out by the first author (NA) in four stages: automatic and manual removal of duplicates, screening of titles and abstracts of search results to remove ineligible studies, reviewing the full-text articles of search results to remove ineligible studies and final paper selection. When it was unclear whether or not an item met eligibility criteria during screening, the reviewer erred on the side of caution and the item was carried into full-text reviewing. The results of the screening and selection process are presented in Fig. 1.

### Data extraction, quality appraisal and analysis

We extracted the following variables from each eligible study into an Excel database: information about the epidemic-prone disease(s) under study, characteristics of study population(s), study aim and objective, concept or definition of risk perception, study design and data collection method(s), results, conclusions and quality of studies.

We assessed the quality of papers using three tools: the Appraisal tool for Cross-Sectional Studies (AXIS tool) for cross-sectional quantitative studies [32], the RATS guidelines for qualitative research [33], and the Mixed Methods Appraisal Tool (MMAT) for mixed method study designs [34]. The quality appraisal tools served to highlight the strengths and weaknesses of the studies to assist in the interpretation of the findings, but no studies were excluded following quality appraisal. We used a narrative meta-synthesis and summary of the evidence to analyse the studies, due to the heterogeneous nature of eligible study designs which did not lend itself to formal meta-analysis. We categorised eligible studies into groups as shown in Table 2.

**Table 2:** Categorisation of eligible studies for analysis

Study population groups (A)	Risk perception dimension groups (B)
Group A1: all studies of risk perceptions among the general population Group A2: all studies of risk perceptions among health professionals *Note: studies which include both public and health professional populations are included in both groups*	Group B1: all studies exploring perceived likelihood as a dimension of risk perception Group B2: all studies exploring perceived susceptibility or vulnerability as a dimension of risk perception Group B3: all studies exploring perceived severity as a dimension of risk perception *Note: studies that explore more than one definition of risk perception were included in more than one group*

Analysis was guided by themes from the SARF [18]. The main premise of the framework is that portrayal of a risk source (e.g. an epidemic-prone disease) and a risk event (e.g. an epidemic and its response) interacts with psychological, social, cultural and institutional processes in ways that may lead to attenuated or amplified risk perceptions [35, 36]. The SARF provides a common terminology for comparing studies from varying disciplines, diseases and populations [37]. We described epidemic risk perceptions levels as ‘high’, ‘moderate’ or ‘low’ according to the scales used in individual eligible studies. We described all factors determined as associated or not associated with epidemic risk perceptions by individual eligible studies, and we organised presentation of factors by components of the SARF. Separately, we described conceptualisations and measurement approaches for each dimension of risk assessment (groups B above).

## Results

### Description of eligible studies (*n* = 56)

We identified fifty-six eligible studies, described in detail in Table 3. Data collection for the studies included in the review occurred between 2008 and 2020, and thirty-seven studies collected data during an active epidemic. Fifty-five studies were cross-sectional, forty-five collected quantitative data, six collected qualitative data and five used mixed methods. The majority of studies were on either Ebola virus disease (EVD) (*n* = 19) or coronavirus disease (COVID-19) (*n* = 18). Three studies compared the risk perception of two or more pathogens in the same population [38–40]. Thirty-three studies measured only one of the four dimensions of risk perception; perceived likelihood of infection was the most frequently reported-on dimension. The main features of the eligible studies are summarised in Table 4.

**Table 3:** Description of eligible studies (n = 56)

Author(s) and year (reference no.)	Country (-ies)	Epidemic-prone disease(s)	Study population(s)	Methods	Results			Quality of study
					Measurements/description of risk perceptions	Factors reported to have an influence on risk perception	Factors reported to have no effect on risk perception	
Abdi et al. 2015 [45]	Kenya	Rift Valley Fever (RVF)	General adult population (pastoralist community)	Cross-sectional study Quantitative data Interviewer-administered questionnaire	Perceived severity: agree 99.2%, disagree 0.8% Perceived likelihood (personal): agree 74%, disagree 26%	None	Gender Area of residence (2 wards in one district were compared, both equally affected by previous RVF outbreaks)	Good
Abou-Abbas et al. 2020 [73]	Lebanon	COVID-19	Health professionals—clinical staff	Cross-sectional study Quantitative data Self-administered questionnaire	32.6% exhibited fears towards working in places where patients suspected of COVID-19 infection are admitted 36.3% reported that they were afraid of treating a patient with COVID-19 infection	None	None	Good
Adhena and Hidru 2020 [64]	Ethiopia	COVID-19	General adult population	Cross-sectional study Quantitative data Interviewer-administered questionnaire	79.2% believed that they are at risk of getting to COVID-19	None	None	Good
Akalu, Ayelign et al. 2020 [57]	Ethiopia	COVID-19	General adult population	Cross-sectional study Quantitative data Interviewer-administered questionnaire	Risk of COVID-19 infection: high 19.8%, moderate 36.1%, low 28.5%, very low 20.5%	None	None	Good
Akram et al. 2015 [40]	Pakistan	Cutaneous leishmaniasis	General adult population	Cross-sectional study Quantitative data Interviewer-administered questionnaire	42% reported that leishmaniasis is more serious than dengue fever	None	None	Poor
Alyousefi et al. 2016 [51]	Yemen	Dengue fever	General adult population (conflict-affected, dengue-endemic area)	Cross-sectional study Quantitative data Interviewer-administered questionnaire	97.7% agree that dengue is a serious disease, 75.5% agree that they are at risk of dengue fever	None	None	Good
Asnakew et al. 2020 [62]	Ethiopia	COVID-19	General adult population	Cross-sectional study Quantitative data Self-administered questionnaire	90.4% perceived that they are susceptible to COVID-19 87.5% perceived that COVID-19 is a serious disease	Marital status, setting/residence, education, income level, occupation, age, family size was associated with perceived susceptibility	Sex, religion had no significant effect on perceived susceptibility Sex, marital status, religion, residence, educational level, income level, occupation, age and family size had no significant effect on perceived seriousness of disease	Acceptable
Ayegbusi et al. 2016 [67]	Nigeria	Ebola virus disease	General adult population (bushmeat handlers [hunters, hawkers, consumers, restaurant owners])	Cross-sectional study Qualitative data In-depth interviews	Some of the respondents expressed some level of anxiety about EVD	The threat posed by EVD to the livelihood of bushmeat sellers, and to well-established use of bushmeat in diet, in spiritual fortification, treatment of disease conditions, seems to be associated with a lower perceived risk The fact that EVD is incurable and no previous outbreak occurred in the country before seems to be associated with higher perceived risk	None	Poor
Bell et al. 2017 [72]	Liberia	Ebola virus disease	Health professionals: community health workers including traditional birth attendants, government community health volunteers, nurses, physician assistants, and midwives	Cross-sectional study Qualitative data Focus group discussions	Participants described a pervasive fear about EVD that permeated their daily lifestyle. Fears about EVD ranged from fear of contracting the disease to a fear of exposing others. Participants were worried for themselves, their families, and their community about contracting or dying from EVD	Fear associated with contracting or spreading the disease due to their positions in the community as healthcare providers; the rapid spread of EVD; the fact that EVD is incurable and not visible; scarce/unavailable personal protective equipment (PPE), non-contact thermometers, handwashing/disinfection facilities/supplies; limited training on how to use PPE and the additional equipment introduced during the response	None	Good
Berman et al. 2017 [58]	Liberia	Ebola virus disease	General adult population: mobile phone users	Cross-sectional study Quantitative data SMS-based survey	50% felt that they were not at all likely to become infected 30% indicated that they were very likely to get infected 20% indicated they were somewhat likely to get infected	Perceived self-efficacy (confidence in their ability to protect themselves)	None	Acceptable
Blum et al. 2014 [39]	Malawi	Typhoid fever	General adult population in villages where typhoid cases had been confirmed	Cross-sectional Qualitative data Free listing exercises In-depth interviews	Typhoid fever was considered the most serious among 23 common illnesses Typhoid was universally viewed as prevalent and extremely dangerous Common diseases, including malaria, were considered comparatively less serious	High risk perception was associated with: Profound economic consequences because those afflicted were unable to farm: The severity of typhoid The continuation of the ongoing outbreak	None	Good
Chaudhary et al. 2020 [74]	Pakistan	COVID-19	Health professionals: clinical and non-clinical oral healthcare workers	Cross-sectional study Quantitative data Self-administered questionnaire	The job risks an exposure to COVID-19 98.5% agree amongst clinical staff, 55% agree amongst non-clinical staff, P-value 0.001 Fear of getting infected by COVID-19: 94.4% agree amongst clinical staff, 80.3% agree amongst non-clinical staff, P-value 0.001 Perceived susceptibility of others: people close to me would be at high risk of getting COVID-19 because of my job 98.5% agree amongst clinical staff, 96.9% agree amongst non-clinical staff I would be concerned for my: Spouse/partner: 77.8% agree amongst clinical staff, 74.3% agree amongst non-clinical staff, non-significant Parents: 59.9% agree amongst clinical staff, 54.5% agree amongst non-clinical staff, non-significant Children: 59.9% agree amongst clinical staff, 66% agree amongst non-clinical staff, non-significant Close friends: 45.9% agree amongst clinical staff, 49.2% agree amongst non-clinical staff, non-significant Work colleagues: 94.1% agree amongst clinical staff, 72.8% agree amongst non-clinical staff, p-value 0.001	None	None	Good
Claude et al. 2019 [107]	Democratic Republic of Congo	Ebola virus disease	General adult population: displaced and non-displaced persons health professionals: nurses and doctors from the study sites	Cross-sectional study Mixed methods Focus group discussions Interviewer-administered questionnaire	The exact measurements of risk perceptions cannot be discerned from the text in the paper. Approximate estimates were discerned from a figure in the paper: 25% perceived high risk, 30% perceived intermediate risk, 45% perceived low risk	None	None	Good
Coulibaly et al. 2013 [108]	Ivory Coast	Pandemic influenza A (H1N1)	Health professionals: doctors, nurses, midwives and support staff in health services	Cross-sectional study Quantitative data Interviewer-administered questionnaire	82.3% Feel at risk of contracting pH1N1 67.6% Fear of becoming infected with pH1N1 22% Fear of becoming influenza-infected at the hospital	None	None	Good
Echoru et al. 2020 [43]	Uganda	COVID-19	General adult population: university lecturers and students	Cross-sectional study Quantitative data Self-administered questionnaire	COVID-19 is dangerous and can kill anyone: 98% said yes amongst lecturers, 98.1% said yes amongst students, difference not significant	None	None	Good
Ekra et al. 2017 [81]	Ivory Coast	Dengue fever	Health professionals—clinical staff	Cross-sectional study Quantitative data Interviewer-administered questionnaire	74% health professionals perceived dengue as a serious illness 43% health professionals perceived the risk of dengue outbreak in Cote d’Ivoire	None	None	Good
Englert et al. 2019 [76]	Uganda	Ebola virus disease Marburg virus disease	Health professionals (clinical and non-clinical workers involved in previous medical responses to outbreaks)	Cross-sectional Qualitative data In-depth interviews	93% of interviewees described being fearful during the EVD outbreak in Gulu All survivors experienced fear, while 75% of the non-infected experienced fear during the EVD outbreak in Bundibugyo In Kabale, 68% of interviewees reported experiencing fear during the Marburg virus outbreak	Alleviated fear: increased PPE availability, prayer, counselling, knowledge of Ebola, vaccine development, earlier diagnostic tools, and a task force with established protocols, continuous education, improved laboratories, robust public education, Marburg-specific training, establishing isolation areas outside main hospital buildings, the presence of role models and experts during the response Increased fear: encountering an infected patient and unusual disease presentations	None	Good
Ernst et al. 2016 [47]	Kenya	Malaria	General adult population in malaria-endemic areas	Cross-sectional study Quantitative data Interviewer-administered questionnaire	Highland areas (seasonal transmission): 97% agree family at risk of malaria, 91% agree malaria is serious, 85% agree children are more at risk than adults Lowland areas (holoendemic transmission): 96% agree family at risk of malaria, 93% agree malaria is serious, 66% agree children are more at risk than adults	None	None	Good
Fatiregun et al. 2012 [78]	Nigeria	Pandemic influenza A (H1N1)	Health professionals—clinical staff	Cross-sectional study Quantitative data Self-administered questionnaire	29.8% perceived their risk of contracting the infection as high	None	None	Good
Ghazi et al. 2020 [63]	Iraq	COVID-19	General adult population	Cross-sectional study Quantitative data Self-administered questionnaire	80.2% perceived COVID-19 as contagious and can lead to death 76.9% perceived COVID-19 as very/seriously dangerous, 20.6% as dangerous, and 2.6% as not dangerous	None	None	Acceptable
Gidado et al. 2015 [59]	Nigeria	Ebola virus disease	General adult population	Cross-sectional study Quantitative data Interviewer-administered questionnaire	61% felt that they cannot contract EVD	Spiritual and divine protection was associated with lower risk perception Self-efficacy (confidence in ability to protect themselves) was associated with higher risk perception	None	Good
Girma et al. 2020 [75]	Ethiopia	COVID-19	Health professionals: clinical and academic staff at university hospitals	Cross-sectional study Quantitative data Self-administered questionnaire	Mean overall risk perception score (out of highest score of 25): 23.59 Mean score (out of highest score of 5): Perceived risk of getting infected with COVID-19: 3.67 Perceived risk of others at work place to get COVID-19: 3.33 Perceived risk of any Ethiopians to get COVID-19: 3.29 Perceived risk of family and friends getting COVID-19: 2.79 Perceived risk of serious COVID-19 illness: 3.48 Perceived risk of death: 2.8 Perceived vulnerability to COVID-19: 4.01 (3.61 HIV/AIDS, 3.87 common cold, 3.32 malaria, 3.64 TB) Perceived severity of COVID-19: 3.63 (3.81 HIV/AIDS, 3.33 common cold, 2.87 malaria, 3.43 TB)	None	None	Acceptable
Girum et al. 2017 [48]	Ethiopia	Malaria	General adult population in malaria-endemic districts	Cross-sectional study Quantitative data Interviewer-administered questionnaire	I think that malaria is a life-threatening disease: 9% disagree, 91% agree I am sure that anyone can get malaria 100% agree In my opinion, children and pregnant women are at higher risk of malaria 2% disagree, 98% agree	None	None	Good
Hakim et al. 2020 [109]	Pakistan	COVID-19	Health professionals—clinical staff	Cross-sectional study Quantitative data Self-administered questionnaire	Perceived likelihood (what do you think is your risk of infection from COVID-19 during your professional duties in the next 30 days?): no risk 1.55% low risk 5.30% medium risk 24.28% high risk 68.87% Perceived likelihood (What do you think is your risk of infection from COVID-19 in your personal life in the next 30 days?): no risk 2.43% low risk 20.97% medium risk 29.14% high risk 47.46%	None	None	Good
Idris et al. 2015 [79]	Nigeria	Ebola virus disease	Health professionals: frontline responders to medical emergencies in rural and urban settings. Includes public and private sector healthcare workers	Cross-sectional study Quantitative data Interviewer-administered questionnaire	Perceived likelihood (risk of being infected): Public sector 17.5% very likely 21.6% not very likely 16% somewhat likely 41.8% not likely at all 3.1% no response Private sector 22.2% very likely 21.6% not very likely 18.6% somewhat likely 30.4% not likely at all 7.2% no response P-value 0.089	None	None	Good
Ilesanmi and Afolabi 2020 [53]	Nigeria	COVID-19	General adult population: urban settings	Cross-sectional study Quantitative data Interviewer-administered questionnaire	26% said they could contract COVID-19 27.5% said it is a deadly disease	None	None	Good
Iliyasu et al. 2015 [77]	Nigeria	Ebola virus disease	General adult population Health professionals—clinical	Cross-sectional study Quantitative data Self-administered questionnaire	Perceived likelihood (moderate to high fear): Kano 78.3%, Bayelsa 64.7%, Calabar 82.2% Perceived severity: 95.8% agree in Kano, 99.2% agree in Calabar	None	None	Acceptable
Iorfa et al. 2020 [69]	Nigeria	COVID-19	General adult population	Cross-sectional study Quantitative data Self-administered questionnaire	Unable to discern from paper	Knowledge of COVID-19 Age (among males)	Age (among females) Gender	Good
Irwin et al. 2017 [110]	Guinea	Ebola virus disease	General adult population	Cross-sectional study Quantitative data Interviewer-administered questionnaire	Perceived likelihood (self-rated risk of contracting Ebola): None or low 82.7% High 17.3%	None	None	Good
Jalloh et al. 2018 [111]	Sierra Leone	Ebola virus disease	General adult population	Cross-sectional study Quantitative data Interviewer-administered questionnaire	72% of respondents perceived Ebola as a threat at one or more levels: to Sierra Leone (69%), their district (58%), their community (53%) or their household (51%)	None	None	Good
Jiang et al. 2016 [60]	Sierra Leone	Ebola virus disease	General adult population: areas at high risk of EVD transmission	Cross-sectional study Quantitative data Interviewer-administered questionnaire	10% of respondents believe that they are at not at risk of contracting Ebola Among 90% of respondents reporting perceived risk of contracting Ebola: 27%, 29%, and 44% reported high, medium, and low perceived risk respectively	Perceived self-efficacy (confidence in ability to protect themselves), occupation, area of residence	Educational level, having ever been to the seaside, getting Ebola information from billboards, and getting Ebola information from brochures	Good
Kabito et al. 2020 [54]	Ethiopia	COVID-19	General adult population	Cross-sectional study Quantitative data Interviewer-administered questionnaire	Prevalence of high-risk perceptions was 23.11% (*n* = 144), 95% CI (19.80–26.43%)	Age, educational status, knowledge of COVID-19	Attitudes towards COVID-19, gender, employment status, monthly income	Good
Kamara et al. 2020 [38]	Sierra Leone	Disease resembling COVID-19 with lower risk of death Disease resembling Ebola virus disease with lower risk of infection	General adult population: two villages with contrasting experiences of EVD outbreak in 2014–15	Cross-sectional Qualitative data An experimental game devised to encourage villagers to talk comparatively about infection risks. Each iteration of the game took about 15 min to complete	Overall, there was a higher preference (52% of all responses) for “mango” (representing EVD). Disease “orange” (representing Covid-19) attracted just over a quarter (27%) of all responses. Players finding no difference between the two disease models accounted for 21% of all responses	A disease’s responsiveness to community infection prevention and control measures Confidence in the possibility of a cure Disease infection risk Disease fatality risk	Gender differences in preferences were not statistically significant	Good
Kaponda et al. 2019 [66]	Malawi	Cholera	General adult population: suspected cholera patients	Cross-sectional study Quantitative data Interviewer-administered questionnaire	Perceived likelihood (total): low 40.7% moderate 34.7% high 24.6% Perceived likelihood (among patients with contaminated water sources at home (200 + cfu/100 ml): 22% reported low risk to themselves and that their communities were well-prepared to respond to future cholera outbreaks	None	None	Good
Kasereka and Hawkes 2019 [50]	Democratic Republic of Congo	Ebola virus disease	General adult population and health professionals residing/working in communities affected by EVD outbreak	Cross-sectional study Mixed methods Focus group discussions Interviewer-administered questionnaire	Affective response: 91% reported they were worried about Ebola	None	None	Acceptable
Kasereka et al. 2019 [70]	Democratic Republic of Congo	Ebola virus disease	General adult population and health professionals residing/working in communities affected by EVD outbreak	Cross-sectional study Quantitative data Interviewer-administered questionnaire	Affective response: worried about Ebola 90% of all respondents; 90% of vaccinated and 90% of unvaccinated respondents Perceived likelihood: Total 43% high 15% intermediate 38% low 3% I don't know Vaccinated 21% high 13% intermediate 64% low 1% I don't know Unvaccinated 64% high 17% intermediate 14% low 5% I don't know *P*-value < 0.001	Vaccination against EVD	None	Acceptable
Khowaja et al. 2011 [71]	Pakistan	Pandemic influenza A (H1N1)	Health professionals: medical students	Cross-sectional study Quantitative data Self-administered questionnaire	62.6% were worried about the current global outbreak of H1N1 40.9% perceived disease as fatal, 29.8% perceived disease as severely dangerous, 15.7% moderately dangerous, 5.1% mildly dangerous, 8.6% unknown	None	None	Acceptable
Mohamed et al. 2017 [112]	Sudan	Ebola virus disease	General adult population: rural residents	Cross-sectional study Quantitative data Interviewer-administered questionnaire	76.3% perceived EVD as so dangerous, 17.5% as dangerous, 3.3% somehow dangerous, 1.1% not dangerous, 0.3% not at all dangerous, 1.5% unknown	None	None	Good
Murele et al. 2014 [113]	Nigeria	Poliomyelitis	General adult population: opinion and religious leaders; parents identified to have persistently refused or accepted vaccination and leaders of community-based organizations	Cross-sectional Qualitative data In-depth interviews	Some of the non-acceptors indicated that nobody was at risk. A few of the respondents mentioned that children were at risk, while others indicated that they do not know who is at risk. Most of the acceptors noted that anyone could fall victim of the virus, but the effects are most typical of children	Vaccine acceptance	None	Poor
Ogoina et al. 2016 [83]	Nigeria	Ebola virus disease	Health professionals: clinical and non-clinical health workers at hospitals	Cross-sectional study Quantitative data Self-administered questionnaire	24.5% rated their fear of EVD 10 out of 10 (highest level of fear) while 19.6% rated their fear 5 out of 10 and 9.8% rated their fear as 1 out of 10. About 40% of respondents expressed fear ratings of EVD of greater or equal to 7 out of 10. There was no professional difference in rating of fear (categories: Doctor–Nurse–Other Health/Paramedical–Non-Medical Health Workers—*P* > 0.05)	None	None	Acceptable
Olowookere et al. 2015 [80]	Nigeria	Ebola virus disease	Health professionals: clinical and non-clinical health workers	Cross-sectional study Quantitative data Self-administered questionnaire	Consider self to be at risk: 39% agree, 42.8% disagree, 18.2% undecided Consider health workers prone to EVD: 75.8% agree, 12.7% disagree, 11.5% undecided	None	None	
Ozioko et al. 2018 [56]	Nigeria	Zoonotic infections	General adult population: bushmeat traders and hunters	Cross-sectional study Quantitative data Interviewer-administered questionnaire	Bushmeat hunters: yes 47.1%, no 52.9% Bushmeat traders: yes 71.4% no 28.6% *P* = 0.36	None	None	Good
Philavong et al. 2020 [65]	Lao	Zoonotic infections	General adult population: market vendors (vegetable, livestock and bushmeat)	Cross-sectional study Quantitative data Interviewer-administered questionnaire	72%of vendors considered that their job did not put their health at risk, highest among bushmeat vendors compared to vegetable or livestock vendors The proportion of vendors who reported that they had “no risk” was higher when asked about their personal risk compared to when they were asked about risk in general, and this was consistent for vegetable vendors (chi-square test, *P* < 0.001), livestock meat vendors (chi-square test, *P* = 0.055) and bushmeat vendors (chi-square test, *P* = 0.0037)	Number of education years Belief in safety and quality of products sold	None	Good
Rizwan et al. 2020 [42]	Pakistan	COVID-19	General adult population: attending a children’s hospital during a lockdown	Cross-sectional study Quantitative data Interviewer-administered questionnaire	How likely you feel you can catch this infection? 59.2% likely/very likely—12.2% neutral—28.6% less likely/very less likely How likely you feel your family members can catch this infection? 52.2% likely/very likely—13.5% neutral—34.3% less likely/very less likely How likely you feel that average Pakistani can suffer from this virus? 58% likely/very likely—19.2% neutral—22.8% less likely/very less likely How likely corona virus infection can be serious? 67.5% likely/very likely—11.2% neutral—21.3% less likely/very less likely What is the chance you have serious complications/death if you get infected? 52.2% likely/very likely—16.1% neutral—31.7% less likely/very less likely What is the chance your family member gets serious infection or die because of corona virus? 37.1% likely/very likely—21% neutral—41.8% less likely/very less likely	Age	None	Acceptable
Schaetti et al. 2013 [41]	Democratic Republic of Congo Kenya Tanzania (Zanzibar)	Cholera	General adult population	Cross-sectional study Quantitative data Interviewer-administered questionnaire	The majority of respondents acknowledge no difference between women and men, adult and children or rich and poor, except in Kenya, where 50.7% report children are more at risk than adults, and 52.2% report the poor are more at risk than the rich Perceived severity: 81.1% DRC, 91.3% Kenya, 96.6% Zanzibar Potential fatality without treatment: 99.7% DRC, 49.9% Kenya, 77.5% Zanzibar (*P*-value < 00.001)	Urban vs. rural setting	Gender	Acceptable
Schmidt-Hellerau et al. 2020 [61]	Sierra Leone	Ebola virus disease	General adult population, including home-based caregivers of suspected Ebola patients (usually family members)	Cross-sectional study Mixed methods Interviewer-administered questionnaire In-depth interviews	43% perceived themselves as being at risk of getting Ebola in the next 6 months	None	None	Good
Sengeh et al. 2020 [114]	Sierra Leone	COVID-19	General adult population	Cross-sectional study Quantitative data Interviewer-administered questionnaire	75% perceived themselves to be at moderate-great risk (95% CI 64.7 to 82.5)	None	None	Good
Shabani et al. 2015 [46]	Tanzania	Rift Valley Fever (RVF)	General adult population: residents in areas that reported the highest number of RVF cases during the 2007 outbreak	Cross-sectional study Quantitative data Interviewer-administered questionnaire	63.2% of respondents reported to be personally at risk of contracting RVF 90.3% agreed that RVF was a serious disease	None	None	Good
Shakeel et al. 2020 [82]	Pakistan	COVID-19	Health professionals—clinical staff	Cross-sectional study Quantitative data Self-administered questionnaire	Perceived severity: 73.42% agree/strongly agree—10.13% disagree/strongly disagree—16.43% neutral	None	None	Good
Tadesse et al. 2020 [115]	Ethiopia	COVID-19	Health professionals—clinical staff: nurses	Cross-sectional study Quantitative data Self-administered questionnaire	Perceived likelihood: 64.6% agree/strongly agree—14.5% neutral, 20.8% disagree/strongly disagree Affective response: 65.2% agree/strongly agree—15.2% neutral, 65.2% disagree/strongly disagree	None	None	Poor
ul Haq et al. 2020 [116]	Pakistan	COVID-19	General adult population	Cross-sectional study Quantitative data Self-administered questionnaire	The majority of the respondents associated the highest risk with COVID-19 (unable to ascertain exact value from the paper)	Urban vs. rural setting	None	Acceptable
Usifoh et al. 2019 [49]	Nigeria	Lassa fever	General adult population: staff and students at the University of Benin, Nigeria	Cross-sectional study Quantitative data Self-administered questionnaire	Perceived likelihood: Staff: 4% no response, 75.7% very seriously, 12% slightly serious, 8.3% not very serious Student: 2% no response, 69.7% very seriously, 20.7% slightly serious, 7.7% not very serious Perceived severity: Staff: 2.7% no response, 83% very serious, 9% slightly serious, 3.7% not very serious, 1.7% not sure Student: 2.3% no response, 76.7% very serious, 14.3% slightly serious, 4% not very serious, 2.7% not sure	None	None	Good
Usuwa et al. 2020 [44]	Nigeria	Lassa fever	General adult population: residents of communities affected by a Lassa fever outbreak	Cross-sectional study Quantitative data Interviewer-administered questionnaire	Perceived susceptibility in the absence of preventive measures: Would you be susceptible: 60.74% certainly yes, 22.7% probably yes, 6.13% neutral, 4.91% probably not, 5.52% certainly not Chances of contracting illness: 41.10% very large chance, 29.75% large chance, 10.74% neutral, 12.27% small chance, 6.13% very small chance Perceived severity of illness: In general: 73.31% very serious, 19.63% serious, 0.92% neutral, 3.37% slightly not serious, 2.76% not serious at all If contracted by respondent: 90.8% very serious, 7.06% serious, 1.23% neutral, 0.31% slightly not serious, 0.61% not serious at all	Knowledge of Lassa fever	None	Good
Winters et al. 2020 [68]	Sierra Leone	Ebola virus disease	General adult population	Longitudinal study (3 repeated cross-sectional surveys, different respondents in each survey) Quantitative data Interviewer-administered questionnaire	Between 50 and 69% of respondents expressed some level of risk perception during the first survey in the four regions. This decreased during the second survey for all regions apart from the Northern Province	Education, area of residence, time of survey in relation to outbreak, gender, age, knowledge of EVD, EVD misconceptions, handwashing, avoiding burials, type and number of information sources	Type of information sources, religion, avoiding physical contact with Ebola-suspects	Good
Xu et al. 2019 [55]	Myanmar	Dengue fever	General adult population: 3 villages with zero, low and high dengue fever incidence	Cross-sectional study Mixed methods Interviewer-administered questionnaire In-depth interviews	Perceived risk (likelihood and severity combined): Total: easy to contract dengue 15.8%, not easy/impossible to contract dengue 5.8%, serious illness 27.8%, deadly disease 24.7%, do not know/no response 68.7% Village 1 (zero incidence): easy to contract dengue 12.9%, not easy/impossible to contract dengue 9.1%, serious illness 27.3%, deadly disease 21.2%, do not know/no response 67.4% Village 2 (low incidence): easy to contract dengue 18.6%, not easy/impossible to contract dengue 4.7%, serious illness 30.2%, deadly disease 27.9%, do not know/no response 60.5% Village 3 (high incidence): easy to contract dengue 19.0%, not easy/impossible to contract dengue 1.2%, serious illness 27.4%, deadly disease 28.6%, do not know/no response 75% Among key informants: higher perception of dengue fever as a serious or deadly disease in villages 2 and 3 compared to village 1	None	None	Good
Xu et al. 2020 [52]	Myanmar	Dengue fever	General adult population: displaced and non-displaced persons	Cross-sectional study Mixed methods Interviewer-administered questionnaire In-depth interviews	Perceived risk (likelihood and severity combined): Total: easy to contract dengue 47.3%, not easy/impossible to contract dengue 42.6%, serious illness 98.4%, deadly disease 98.1%, do not know/no response 10.1% IDP: easy to contract dengue 38.7%, not easy/impossible to contract dengue 51.1%, serious illness 97.8%, deadly disease 97.8%, do not know/no response 10.2% Host community: easy to contract dengue 57%, not easy/impossible to contract dengue 33.1%, serious illness 99.2%, deadly disease 98.3%, do not know/no response 9.9% Higher risk perception among key informants in camp compared to health workers interviewed	None	None	Good

**Table 4:** Main features of eligible studies

Characteristic	Number of studies
Country	
Democratic Republic of Congo	4^a^
Ethiopia	5
Guinea	1
Iraq	1
Ivory Coast	2
Kenya	3^a^
Lao	1
Lebanon	1
Liberia	2
Malawi	2
Myanmar	2
Nigeria	13
Pakistan	7
Sierra Leone	6
Sudan	1
Tanzania	2^a^
Uganda	2
Yemen	1
Epidemic-prone disease	
Ebola virus disease	19^b^
COVID-19	18^b^
Dengue fever	4^b^
Pandemic influenza A (H1N1)	3
Cholera	2
Lassa fever	2
Malaria	2
Rift valley fever	2
Zoonotic infections	2
Cutaneous leishmaniasis	1^b^
Marburg virus disease	1
Poliomyelitis	1
Typhoid fever	1^~^
Active epidemic during data collection?	
Yes	19
No	37
Type of study population	
General population (15 years or older)	40
General population: cases with disease under study	1
Clinical health professionals	10
Other health professionals	9
Data collection methods	
Self-administered questionnaire (in-person or online)	17
Interviewer administered questionnaire	32
Focus group discussion	2
Semi-structured interviews	7
Free listing	1
Experimental fame	1
SMS-based survey	1
Number of dimensions of risk perception reported on	
One	33
Two	36
Three	3
Dimensions of risk perception reported on	
Perceived likelihood	36
Perceived severity	27
Perceived susceptibility	8
Affective risk perception	14
Conceptual framework used	
No framework	36
Knowledge, attitudes and practices (kap)	10
Health belief model	3
Explanatory model interview catalogue	1
Moderated mediation model	1
Ideation metatheory	1
Social process theory	1
Weberian social action theory	1
Original framework developed by authors	1
Method for measuring/assessing risk perception	
Likert- or Likert-type scale	34
Dichotomous question (yes/no; agree/disagree)	11
Open-ended question	3
Comparison of two diseases	2
Ranking of diseases	1
Comparison of vulnerability of two population groups	1
Score against pre-determined ‘correct’ risk perception defined by author	1
Unable to ascertain	6

Note that totals may exceed the number of eligible studies (*n* = 56) as some studies explored more than one category

^a^Of which one is multi-country

^b^Includes comparison with other pathogens within a study

Below, we summarise themes related to risk perception and factors influencing risk perceptions, for the general population and health professionals separately.

### Epidemic risk perceptions among the general population (*n* = 41)

Forty-one studies included measurement or description of risk perception of epidemic-prone diseases among non-expert populations. Regardless of countries, diseases under study or whether there was an active outbreak at the time of the study, participants tended to report a high perceived severity of epidemic-prone diseases, generally above the midpoints of severity scales used by researchers [39, 41–52]. In contrast, perceived personal likelihood of contracting an epidemic-prone infection varied across studies, from low [52–56] to moderate [57–61] and high [42, 43, 47–49, 51, 62–64]. This variation persisted across countries, diseases under study and whether there was an active outbreak at the time of the study. For example, two COVID-19 studies in Ethiopia in 2020 reported contrasting levels of perceived likelihood [54, 62]. However, perceived likelihood of personally contracting infections tended to be lower than perceived severity in studies that measured both aspects of perceived severity [45, 46, 51, 52].

Another theme was a pattern of perceiving risk of epidemic-prone diseases to others as higher than to self, and that the risk to distant individuals or communities is higher than to closer ones. For example, in a study in Sierra Leone, participants perceived the threat of EVD as highest for the country, followed by the district, community then household [50]. Another study of perceived zoonotic infection risks among market vendors also showed a perceived lower risk of infection to self, compared to the rest of the general population [65].

Among groups with a higher risk of exposure to epidemic-prone diseases, perceived likelihood of infections appeared lower than among the general population. For example, among suspected cholera patients, only a quarter thought they were at high risk of contracting cholera again—even where researchers found high levels of water contamination in their households at the time of the study [66]. Similarly, two studies showed that bushmeat hunters and vendors had reduced perceived likelihood of EVD compared to bushmeat consumers [67], and of zoonotic infections compared to vendors selling livestock or vegetables [65].

Participants also perceived some populations groups as more susceptible to risks of epidemic-prone diseases than others. For example, both internally-displaced persons (IDP) and non-displaced host communities perceived IDPs as more vulnerable to dengue fever [52]. Similarly, adult community members perceived pregnant women and children to malaria compared to others in malaria-endemic regions [48].

#### Information sources and channels

Respondents who acknowledged the risk of acquiring EVD in the next 6 months during an outbreak were more likely to acquire information from their community (e.g. community leaders, friends and relatives) or new media (e.g. internet, text messages), and accessed three or more information sources. Television, radio, house visits by health workers and government campaigns, and using two or less information sources appeared to have no influence on perceived risk [68]. Two studies showed inconsistent effects of newspapers, brochures and billboards on risk perception [60, 68]. Previous community experience of disease [55] and exposure to a new and unfamiliar disease [67] were associated with increased risk perception.

#### Individual factors

Demographic factors showed inconsistent influences on risk perception across countries and diseases. Education level [54, 60, 62, 65, 68], disease-specific knowledge [44, 54, 68, 69], rural or urban residence [41, 42, 62], marital status [54, 62], income level [54, 62], gender [41, 54, 62, 68, 69] and age [42, 54, 62, 68, 69], variably showed positive, negative or no association with epidemic risk perception across different studies. Larger family size [62] and certain occupations [60, 62] were associated with increased perceived risk in two studies. By contrast, employment status [54] was not associated with risk perception. While no specific religion was associated with risk perception [41, 62], belief in divine or spiritual protection against harm appeared to reduce perceived EVD risk [59, 67].

#### Disease attributes

Disease case fatality ratios and infection risks seemed to influence risk perception, indicating the role of numeracy skills [39]. The phase of an outbreak also seemed influential: an ongoing outbreak of typhoid fever was associated with a grave concern that cases would continue to increase [39], while the likelihood of acknowledging the risk of acquiring infection decreased as an EVD outbreak progressed [68]. Some disease attributes were associated with an increased risk perception among participants, specifically diseases perceived as hard to control through community infection control measures [38], unfamiliar diseases [67], and severe diseases [39, 67]. Participants cited multiple features of evident disease severity, such as rapid spread, unpredictable nature, severe or debilitating symptoms, ineffectiveness of traditional or biomedical treatments and the profound economic consequences of a debilitating illness [39, 67].

#### Health protective behaviours

Three studies explored the association between risk perception and a person’s belief in their ability to protect themselves from EVD, and concluded that a higher self-efficacy is associated with lower perceived risk and vice versa [58–60]. Another study found that vaccination against EVD lowered perceived likelihood and alleviated worry [70]. However, the relationship between risk perception and protective behaviours against EVD was not consistent; for example, one study reported that while handwashing had a positive association with risk perception, avoiding burials was negatively associated with risk perception, and avoiding physical contact with a suspected EVD case not associated with risk perception [68].

#### The sociocultural context

Among vendors, familiarity with, knowledge of and preference of a vendor’s own products, was associated with a reduced perceived risk of zoonotic infections. In one study of perceived zoonotic infection risks among market vendors in Lao, vegetable vendors reported that their products were “organic”, “healthy” and “natural”, and livestock meat vendors mentioned that their meat was mainly sourced from slaughterhouses with robust veterinary control [65]. For some bushmeat vendors, not being involved in the hunting and killing of wild animals seemed to be perceived as reducing their risk of zoonotic infections [65]. Another study amongst bushmeat handlers in Nigeria reported a low perceived risk of EVD and questioned the plausibility that well-established traditional uses of bushmeat, such as diet, spiritual fortification and treatment of disease conditions, could be risky [67].

In a multi-country study of the sociocultural features of cholera, the authors observed that in Kenya, respondents perceived women and children as more vulnerable to cholera compared to the general population, and suggested that this may be due to greater cultural sensitivity to vulnerability amongst the study participants, and a tendency to generalize the vulnerability of already-vulnerable population groups to include susceptibility to disease [41].

Table 5 summarizes the factors reported by eligible studies and their influence of epidemic risk perceptions among the general population, by element of the SARF.

**Table 5:** Factors reported and their influence on epidemic risk perceptions, by element of the SARF

Category	SARF element			
	Information sources and channels	Social stations	Individual stations	Institutional and social behaviour
	*n* = 4	*n* = 0	*n* = 14	*n* = 0
General population				
Factors reported to have an influence on risk perception	Access to three or more information sources Access to community information sources (e.g. community leaders, friends and relatives) Access to new media (e.g. internet, text messages)	–	Family size Occupation Belief in divine or spiritual protection against harm Disease case fatality ratios and infection risks Phase of an outbreak Disease’s responsiveness to community infection control measures Familiarity/novelty of disease Disease severity Personal self-efficacy Vaccination Among some high-risk occupational groups: knowledge of and preference of a person’s services/products Cultural sensitivities or tendencies	–
Factors reported to not have an influence on risk perception	–	–	Employment status Religion	–
Factors inconsistently influencing risk perceptions	Previous community experience of disease Newspapers, brochures and billboards as epidemic information sources	–	Education level Disease-specific knowledge Rural or urban residence Marital status Income level Gender Age Compliance with protective behaviours	–

	*n* = 2	*n* = 0	*n* = 2	*n* = 3
Health professionals				
Factors reported to have an influence on risk perception	Disease-specific knowledge e.g. through training	–	Familiarity with clinical presentation Speed of disease spread Predictability of outbreak Availability of a pharmacological cure Possibility of encounters with infected patients Witnessing deaths among colleagues Potential to spread infection to others in the community	Efficacy of health system response Access to vaccination Existence of vaccine research
Factors reported to not have an influence on risk perception	–	–	–	–
Factors inconsistently influencing risk perceptions	–	–	–	–

–: blank; SARF: social amplification of risk framework

### Epidemic risk perceptions among health professionals (*n* = 19)

Studies reporting on health professionals’ epidemic risk perceptions focused on how they perceived their own risk rather than the risk to communities they served. All studies but one concerned epidemic-prone infections that can readily be acquired in a healthcare setting: COVID-19, EVD, Marburg virus and pandemic influenza A (H1N1). Eighteen studies included clinical staff, six included non-clinical health facility staff, and three studies included community-based health workers. One study solely included medical students [71].

Health professionals generally reported high perceived likelihood and susceptibility to infections [72–77]. In three studies, however, only about a third considered themselves to be at risk [78–80]. All three studies were conducted during an active outbreak in Nigeria: two related to EVD, and one related to H1N1. Health professionals generally reported a high perceived severity of epidemic-prone diseases [71, 77, 81, 82], including high perceived disease severity should they acquire the infection themselves [72, 75].

When comparing clinical to non-clinical staff, the results of perceived risks were inconclusive. One study reported that clinical staff had higher perceived risk than non-clinical staff [74], while another study reported no significant difference in fear ratings of doctors, nurses, paramedical staff and non-clinical workers [83]. Similarly, the review findings were inconclusive with regards to whether health workers rated the risk to themselves as higher or lower than that of other health workers [75, 80].

#### Information sources and channels

Two studies reported that acquiring disease-specific knowledge, for example through training, alleviated fear among health workers and reduced their perceived vulnerability to EVD infection [72, 76].

#### Disease attributes

Health professionals reported disease attributes that increased their fear, specifically unusual clinical presentations [76], the rapid spread and unpredictable nature of an outbreak, and diseases without a pharmacological cure, such as EVD [72].

Within the clinical environment, health professionals reported that encounters with infected patients [76], witnessing colleagues die [76], and the potential to spread infection to others in the community [72] all increased their fear.

#### Institutional response

Health professionals reported a number of factors associated with the health system response that influenced perceived risk. These included indicators of institutional efficacy that alleviate fear, such as clear protocols and operating procedures for patient triage and isolation, the presence of experts and role models early on in the response, availability of personal protective equipment (PPE), rapid and early diagnostic tools, non-contact thermometers and sufficient handwashing and disinfection supplies and facilities [72, 76]. The studies also reported that access to vaccination [70], and vaccine research and development for diseases such as EVD [76] reduce perceived susceptibility among health workers.

Table 5 summarizes the factors reported by eligible studies and their influence of epidemic risk perceptions among health professionals, by element of the SARF.

### Conceptualisation and measurement of epidemic risk perceptions (*n* = 56)

Studies applied variable conceptualisations of risk perception, as reflected in data collection instruments and wording of questions. For perceived likelihood, thirty-four studies conceived of this as research participants themselves contracting infection, while other studies asked participants about the likelihood of others getting infected. Twenty-seven studies used the term “risk” while other studies asked respondents about “possibility”, “probability”, or “chance”. Only three studies provided time windows in their questions, for example, risk over the next 6 months. Perceived susceptibility was conceptualised by two studies as the likelihood of contracting infections in the absence of preventive measures, and by another two studies as the comparative susceptibility among groups. For perceived severity, nineteen studies operationalised this as ‘seriousness’ or ‘dangerousness’ of the disease. Other studies asked participants about the likelihood of certain outcomes (recovery, survival, severe illness, death) should they be infected. Finally, for affective perception, thirteen studies measured ‘fear’ or ‘worry’. Two studies asked the research participant about emotions (e.g. fear or worry) towards their family members, and one study asked participants about the threat to their community, district and country.

Likert-type or Likert scales, ranging from 3- to 10-point scales, were by far the most commonly-used tool for risk perceptions across all conceptualisations. However, the use of neutral or ‘don’t know’ categories was inconsistent. Furthermore, some scales measured degrees or levels of “risk” while others measured respondents’ levels of agreement with statements. This heterogeneity in measurement modalities, measured aspects and wording limited comparability between studies. Furthermore, for several papers we could not ascertain the measurement method used.

Details of the conceptual frameworks, definitions and measurement of risk perception used by eligible studies are provided in Table 6.

**Table 6:** Conceptualisations, definitions and measurements of risk perception in eligible studies (*n* = 56)

Author(s) and year (reference no.)	Country (-ies)	Epidemic-prone disease (s) under study	Study population(s)	Study aim	Conceptual framework	Definition of risk perception	Methods (study design, type of data collected, data collection method(s), methods for assessing/measuring risk perception)
Abdi et al. 2015 [45]	Kenya	Rift Valley Fever (RVF)	General adult population	To assess the knowledge, attitudes, and practices regarding RVF among a pastoralist community	KAP	Perceived severity (RVF is a dangerous disease) Perceived likelihood (you are at a risk of RVF infection)	Cross-sectional study; quantitative data; interviewer-administered questionnaire; 5-point Likert-type scale
Abou-Abbas et al. 2020 [73]	Lebanon	COVID-19	Health professionals	To assess the knowledge and practices of physicians regarding COVID-19, and to evaluate their fear towards COVID-19 and their perceptions regarding policies/actions implemented by the government and their health care settings in handling COVID-19 pandemic	None	Affective response (fear towards COVID-19)	Cross-sectional study; quantitative data; self-administered questionnaire; 3-point Likert-type scale
Adhena and Hidru 2020 [64]	Ethiopia	COVID-19	General adult population	To assess the knowledge, attitude, and practice of high-risk age groups towards COVID-19 prevention and control	KAP	Perceived likelihood (think he/she is at risk of getting sick with the new coronavirus)	Cross-sectional study; quantitative data; interviewer-administered questionnaire; yes/no response options
Akalu et al. 2020 [57]	Ethiopia	COVID-19	General adult population	To determine the knowledge, attitudes, and practices towards COVID-19 and associated factors of poor knowledge and practice among chronic disease patients	KAP	Perceived likelihood (risk of infection with COVID-19)	Cross-sectional study; quantitative data; interviewer-administered questionnaire; 4-point Likert-type scale
Akram et al. 2015 [40]	Pakistan	Cutaneous leishmaniasis	General adult population	To assess the level of knowledge, attitude and practices of the community related to cutaneous leishmaniasis	KAP	Perceived severity (seriousness of the disease as compared to dengue fever)	Cross-sectional study; quantitative data; interviewer-administered questionnaire; choice of two comparative statements: Leishmaniasis is more serious than dengue fever OR dengue fever is more serious than leishmaniasis
Alyousefi et al. 2016 [51]	Yemen	Dengue fever	General adult population	To describe the knowledge, attitudes, and practices of local urban communities towards dengue fever	KAP	Perceived severity (dengue fever is a serious disease) Perceived likelihood (I am at risk of dengue fever)	Cross-sectional study; quantitative data; interviewer-administered questionnaire; 4-point Likert-type scale
Asnakew et al. 2020 [62]	Ethiopia	COVID-19	General adult population	To assess the community’s level of risk perception of COVID-19, precautionary behaviour, and intention to comply with the nonpharmaceutical preventive measures	None	Perceived likelihood (likelihood of being infected with the COVID-19 at any point in the future/likelihood of families or friends will be infected with the COVID-19 at any point in the future/likelihood they will contract COVID-19 from families or friends) Perceived severity (subjective: chance of having recovering from COVID-19/chance of surviving if infected with COVID-19/chance of having no symptoms if infected with COVID-19/the chance of having mild disease if infected with COVID-19 i.e. e.g. can go about daily tasks normally)—(objective: perceived seriousness of COVID-19) Affective perception (their level of worry due to COVID-19)	Cross-sectional study; quantitative data; self-administered questionnaire; 5-point Likert-type scale
Ayegbusi et al. 2016 [67]	Nigeria	Ebola virus disease	General adult population	To examine the perception of the target population on their vulnerability to EVD and the prevention practices they observe to guard against being infected	Weberian social action theory	Perceived likelihood (to be infected to COVID-19)	Cross-sectional study; qualitative data; in-depth interviews; cannot be discerned from paper
Bell et al. 2017 [72]	Liberia	Ebola virus disease	Health professionals	To explore healthcare providers’ perceptions and reactions to the EVD epidemic	None	Affective perception (tell us about your biggest fears for yourself as a community health worker because of Ebola)	Cross-sectional study; qualitative data; semi-structured focus group discussions; open-ended question
Berman et al. 2017 [58]	Liberia	Ebola virus disease	General adult population	To rapidly collect information from communities on the front lines of the outbreak	The ideation metatheory	Perceived likelihood (how likely are you to be infected?)	Cross-sectional study; quantitative data; SMS-based survey; 3-point Likert-type scale
Blum et al. 2014 [39]	Malawi	Typhoid fever	General adult population	To investigate factors associated with the acceptability of typhoid vaccine in response to this ongoing typhoid outbreak	None	Perceived severity (perceived severity of typhoid compared with other common illnesses)	Cross-sectional; qualitative data; freelisting exercises, in-depth interviews; free listing and open-ended questions
Chaudhary et al. 2020 [74]	Pakistan	COVID-19	Health professionals	To evaluate/contrast the clinical and non-clinical oral healthcare workers’ concerns, perceived impact, and preparedness for the COVID-19 pandemic	None	Perceived susceptibility to infection (the job risks an exposure to COVID-19) Affective response (fear of getting infected by COVID-19)	Cross-sectional study; quantitative data; self-administered questionnaire; 6-point Likert-type scale
Claude et al. 2019 [107]	Democratic Republic of Congo	Ebola virus disease	General adult population	To explore social resistance to EVD control efforts during the current persistent outbreak	None	Perceived likelihood (participants were asked to identify whether they felt they were at high, intermediate or low risk of contracting EVD)	Cross-sectional study; mixed methods; focus group discussions, interviewer-administered questionnaire; 3-point Likert-type scale
Coulibaly et al. 2013 [108]	Ivory Coast	Pandemic influenza A (H1N1)	Health professionals	To determine health professionals’ level of knowledge about the influenza pandemic and their willingness to be vaccinated	None	Perceived likelihood (feel at risk of contracting pH1N1) Affective response (fear of becoming infected with pH1N1 AND fear of becoming influenza infected at the hospital)	Cross-sectional study; quantitative data; interviewer-administered questionnaire; yes/no response options
Echoru et al. 2020 [43]	Uganda	COVID-19	General adult population	To determine the knowledge, attitudes, and preparedness/practices of lecturers and students in the fight against COVID-19	None	Perceived severity (COVID-19 is dangerous and can kill) Perceived likelihood (anyone can get COVID-19)	Cross-sectional study; quantitative data; self-administered questionnaire; yes/no response options
Ekra et al. 2017 [81]	Ivory Coast	Dengue fever	Health professionals	To identify the determinants of good practices in the diagnosis of dengue among healthcare workers	None	Perceived severity (perception of the seriousness of the disease) Perceived likelihood (their perception of the fact that Cote d’Ivoire can be at risk of dengue)	Cross-sectional study; quantitative data; interviewer-administered questionnaire; yes/no response options
Englert et al. 2019 [76]	Uganda	Ebola virus disease Marburg virus disease	Health professionals	To describe the perspectives and actions of health workers in three filovirus outbreaks between 2000 and 2012	The social process theory	Affective perception (how concerned were you for your own well-being?—did you ever experience fear, anxiety or depression from the outbreaks?)	Cross-sectional; qualitative data; in-depth interviews; Open-ended question
Ernst et al. 2016 [47]	Kenya	Malaria	General adult population	To determine factors associated with household-level ownership of bed nets factors associated with not using all available bed nets	The health belief model	Perceived likelihood (family at risk of malaria) Perceived severity (malaria is serious) Perceived susceptibility (children are more at risk than adults)	Cross-sectional study; quantitative data; interviewer-administered questionnaire; unable to ascertain from paper
Fatiregun et al. 2012 [78]	Nigeria	Pandemic influenza A (H1N1)	Health professionals	To determine the willingness of doctors and nurses working in health facilities to receive the pandemic A vaccine and to identify factors associated with their willingness to receive the vaccination	None	Perceived likelihood (perception of risk of contracting the infection)	Cross-sectional study; quantitative data; self-administered questionnaire; risk perception of contracting infection was scored based on 13 questions from the risk perception section. Each correct perception was awarded one point while the wrong perception was awarded no points. Scores < 7 were categorised as low risk perception, and those with and scores ≥ 7 were categorised as high risk perception
Ghazi et al. 2020 [63]	Iraq	COVID-19	General adult population	To assess knowledge, attitude, and practice toward COVID-19	KAP	Perceived severity (I think COVID-19 is contagious and can lead to death/cannot lead to death AND I feel COVID-19 is dangerous/very dangerous/seriously dangerous/not dangerous)	Cross-sectional study; quantitative data; self-administered questionnaire; choice of two comparative statements: contagious and cannot lead to death OR contagious and can lead to death, 4-point Likert-type scale
Gidado et al. 2015 [59]	Nigeria	Ebola virus disease	General adult population	To assess public knowledge, perception and adequacy of information on EVD	None	Perceived likelihood (risk of contracting infection)	Cross-sectional study; quantitative data; interviewer-administered questionnaire; unable to ascertain from paper
Girma et al. 2020 [75]	Ethiopia	COVID-19	Health professionals	To assess health professionals’ risk perception and their precautionary behavioural responses	None	Perceived likelihood (perception of risk of contracting the infection) Perceived severity Perceived susceptibility (perceived vulnerability to infection, and respondents’ self-efficacy)	Cross-sectional study; quantitative data; self-administered questionnaire; 5-point Likert-type scale
Girum et al. 2017 [48]	Ethiopia	Malaria	General adult population	To identify factors affecting prevention and control of malaria	None	Perceived severity (I think that malaria is a life-threatening disease) Perceived likelihood (I am sure that anyone can get malaria) Perceived susceptibility (In my opinion, children and pregnant women are at higher risk of malaria)	Cross-sectional study; quantitative data; interviewer-administered questionnaire; 4-point Likert-type scale
Hakim et al. 2020 [109]	Pakistan	COVID-19	Health professionals	To assess self-reported access to PPE, whether adequate information was provided about the use of PPE, COVID-19 risk perceptions, and the ability to perform donning and doffing of PPE	None	Perceived likelihood (risk perception of contracting the disease during professional duty and daily life)	Cross-sectional study; quantitative data; self-administered questionnaire; 4-point Likert-type scale
Idris et al. 2015 [79]	Nigeria	Ebola virus disease	Health professionals	To determine and compare what two subgroups of the health community know, what their beliefs are, and what their current practices are with regards to EVD	None	Perceived likelihood (risk of contracting infection)	Cross-sectional study; quantitative data; interviewer-administered questionnaire; 5-point Likert-type scale
Ilesanmi and Afolabi 2020 [53]	Nigeria	COVID-19	General adult population	To assess the perception and practices of community members in urban areas regarding COVID-19	None	Perceived likelihood (risk of contracting infection) perceived severity (It is a deadly disease)	Cross-sectional study; quantitative data; interviewer-administered questionnaire; yes/no response options
Iliyasu et al. 2015 [77]	Nigeria	Ebola virus disease	General adult population, health professionals	To ascertain the knowledge, attitude and practice of EVD in three states of Nigeria	KAP	Affective perception (fear of getting EVD) Perceived severity (Ebola is a serious disease)	Cross-sectional study; quantitative data; self-administered questionnaire; perceived likelihood: 10-point Likert-type scale, perceived severity: 4-point Likert-type scale
Iorfa et al. 2020 [69]	Nigeria	COVID-19	General adult population	To explore the relationship between COVID-19 knowledge, risk perception, and precautionary behaviour, and to determine whether this relationship differed for men and women	The moderated mediation model	Affective perception (worry about contracting COVID-19)	Cross-sectional study; quantitative data; self-administered questionnaire; 7-point Likert-type scale
Irwin et al. 2017 [110]	Guinea	Ebola virus disease	General adult population	To assess attitudes about Ebola vaccines	None	Perceived likelihood (self-rated risk of contracting Ebola)	Cross-sectional study; quantitative data; interviewer-administered questionnaire; 3-point Likert-type scale
Jalloh et al. 2018 [111]	Sierra Leone	Ebola virus disease	General adult population	To estimate prevalence of mental health symptoms and factors associated with having symptoms	None	Affective perception (perceived threat of Ebola to country, district, community, household)	Cross-sectional study; quantitative data; interviewer-administered questionnaire; 4-point Likert-type scale
Jiang et al. 2016 [60]	Sierra Leone	Ebola virus disease	General adult population	To understand the knowledge, attitudes, practices, and perceived risk of EVD among the public	None	Perceived likelihood (risk of contracting infection)	Cross-sectional study; quantitative data; interviewer-administered questionnaire; 3-point Likert-type scale
Kabito et al. 2020 [54]	Ethiopia	COVID-19	General adult population	To analyse the prevalence and factors associated with risk perception of COVID-19 infections	None	Perceived susceptibility (how likely one considered oneself (his/her families) would be infected with COVID-19 if no preventive measure will be taken) Perceived severity (proxied by how one rated the seriousness of symptoms caused by COVID-19, their perceived chance of having COVID-19 cured and that of survival if infected)	Cross-sectional study; quantitative data; interviewer-administered questionnaire; 5-point Likert-type scale
Kamara et al. 2020 [38]	Sierra Leone	Disease resembling COVID-19 Disease resembling Ebola virus disease	General adult population	To gain insight into how rural people faced with Covid-19 assess epidemic infection risks	None	perceived likelihood (chance of being infected or not) Perceived severity (chance of dying or surviving the diseases)	Cross-sectional; Qualitative data; An experimental game devised to encourage villagers to talk comparatively about infection risks; preference for one of two scenarios of diseases with likelihood of infection and death
Kaponda et al. 2019 [66]	Malawi	Cholera	General adult population	To investigate drinking water source quality compared with water treatment, risk perception and cholera knowledge for patients who had reported to a health centre for treatment	None	Perceived likelihood (personal risk for contracting cholera in the future)	Cross-sectional study; quantitative data; interviewer-administered questionnaire; 3-point Likert-type scale
Kasereka and Hawkes 2019 [50]	Democratic Republic of Congo	Ebola virus disease	General adult population, health professionals	To probe community beliefs around Ebola and its origins	None	Affective perception (‘Are you worried about Ebola?’)	Cross-sectional study; mixed methods; focus group discussions, Interviewer-administered questionnaire; yes/no response options
Kasereka et al. 2019 [70]	Democratic Republic of Congo	Ebola virus disease	General adult population	To describe patient-reported side effect profiles and vaccination experiences, attitudes towards the vaccine, as well as desires for personal and community vaccination	None	Affective perception (‘Are you worried about Ebola?’) Perceived likelihood (personal risk of contracting EVD)	Cross-sectional study; quantitative data; interviewer-administered questionnaire; affective perception: yes/no response options, perceived likelihood: 4-point Likert-type scale
Khowaja et al. 2011 [71]	Pakistan	Pandemic influenza A (H1N1)	Health professionals	To assess student awareness of the H1N1 pandemic	None	Affective perception (worried about current global outbreak) Perceived severity (severity of disease)	Cross-sectional study; Quantitative data; Self-administered questionnaire; 5-point Likert-type scale
Mohamed et al. 2017 [112]	Sudan	Ebola virus disease	General adult population	To explore the knowledge, attitude and practices of rural residents in Sudan regarding Ebola haemorrhagic fever	None	Perceived severity (severity of disease)	Cross-sectional study; quantitative data; interviewer-administered questionnaire; 6-point Likert-type scale
Murele et al. 2014 [113]	Nigeria	Poliomyelitis	General adult population	To explore and document the perceptions of vaccine among care givers who accept or refuse the immunization of their children against polio virus	The health belief model	Perceived susceptibility to polio virus infection	Cross-sectional; qualitative data; in-depth interviews; open-ended question
Ogoina et al. 2016 [83]	Nigeria	Ebola virus disease	Health professionals	To report the opinions and behaviours of healthcare workers during an EVD outbreak	None	Affective perception (affective response: “how would you rate your fear of Ebola?”)	Cross-sectional study; quantitative data; self-administered questionnaire;10-point Likert-type scale
Olowookere et al. 2015 [80]	Nigeria	Ebola virus disease	Health professionals	To assess the preparedness of health workers in the control and management of EVD	None	Perceived susceptibility (of self: Consider self to be at risk—of others: health workers are prone to having EVD)	Cross-sectional study; quantitative data; self-administered questionnaire; 3-point Likert-type scale
Ozioko et al. 2018 [56]	Nigeria	Zoonotic infections	General adult population	To evaluate bushmeat dealers’ knowledge and attitudes about zoonotic infections and the risk of transmission to humans	None	Perceived likelihood (contracting a work-related zoonosis)	Cross-sectional study; quantitative data; interviewer-administered questionnaire; yes/no response options
Philavong et al. 2020 [65]	Lao	Zoonotic infections	Ge#neral adult population	To establish baseline characteristics of market traders (demography, geographical origins) and their perception, behaviours and practices in regard to disease risk in markets	None	Perceived likelihood (risk to self of contracting disease from items sold—risk to others in same vendor group from items sold—risk of disease transmission due to occupation)	Cross-sectional study; quantitative data; interviewer-administered questionnaire; yes/no/unsure response options
Rizwan et al. 2020 [42]	Pakistan	COVID-19	General adult population	To determine the knowledge, risk perception and behavioural response of COVID-19		Perceived likelihood (risk of contracting infection to self—to family member—to average Pakistani) Perceived severity (of disease in general—of disease if personally contracted infection—of disease if family member contracted infection)	Cross-sectional study; quantitative data; interviewer-administered questionnaire; 5-point Likert-type scale
Schaetti et al. 2013 [41]	Democratic Republic of Congo Kenya Tanzania (Zanzibar)	Cholera	General adult population	To review and systematically compare local cholera-related recognition, risk perceptions, experience, and meaning in endemic settings	Explanatory Model Interview Catalogue framework	Perceived likelihood (risk to different population groups) Perceived severity (perceived seriousness of cholera—potential fatality of cholera)	Cross-sectional study; quantitative data; interviewer-administered questionnaire; Perceived likelihood: categorical response options + open-ended question for justification of choice (males or females? adults or children? rich or poor people?), Perceived severity: 4-point Likert-type scale + open-ended question for justification of choice
Schmidt-Hellerau et al. 2020 [61]	Sierra Leone	Ebola virus disease	General adult population	To obtain a contextual understanding of intended and reported protective measures when caring for suspected Ebola patients at home during an outbreak	KAP	Perceived likelihood (perceived risk of contracting EVD in the next 6 months)	Cross-sectional study; mixed methods; interviewer-administered questionnaire, in-depth interviews; 5-point Likert-type scale
Sengeh et al. 2020 [114]	Sierra Leone	COVID-19	General adult population	To assess the public’s knowledge, attitudes and practices about the novel coronavirus	KAP	Perceived likelihood (risk of contracting infection in the next 6 months)	Cross-sectional study; quantitative data; interviewer-administered questionnaire; unable to ascertain from paper
Shabani et al. 2015 [46]	Tanzania	Rift Valley Fever (RVF)	General adult population	To determine perceived risk of RVF among community members	None	Perceived likelihood (perceived risk of contracting RVF) Perceived severity (RVF is a serious disease)	Cross-sectional study; quantitative data; interviewer-administered questionnaire; 5-point Likert-type scale
Shakeel et al. 2020 [82]	Pakistan	COVID-19	Health professionals	To evaluate the knowledge, attitude, and precautionary practices of healthcare providers towards COVID-19	None	Perceived severity (COVID-19 is a dangerous disease)	Cross-sectional study; quantitative data; self-administered questionnaire; 5-point Likert-type scale
Tadesse et al. 2020 [115]	Ethiopia	COVID-19	Health professionals	To investigate knowledge, attitudes and practices, and psychological response towards COVID-19 among nurses	None	Perceived likelihood (risk of infection to self—risk of infection to family members) Affective response (worried that one of your family members will get an infection)	Cross-sectional study; quantitative data; self-administered questionnaire; 5-point Likert-type scale
ul Haq et al. 2020 [116]	Pakistan	COVID-19	General adult population	To assess the knowledge of the general public both rural and urban about COVID-19; to determine precautionary measures taken by rural and urban people to avoid COVID-19; to determine the factors affecting precautionary measures; to assess the behaviour of rural and urban people towards COVID-19; to check the availability and affordability of essential protective items for rural and urban people	Developed by authors	Perceived severity (how risky is COVID-19 in your view?)	Cross-sectional study; quantitative data; self-administered questionnaire; 5-point Likert-type scale
Usifoh et al. 2019 [49]	Nigeria	Lassa fever	General adult population	To assess the perceived stigmatization associated with LF outbreaks among university staff and students	None	Perceived likelihood (possibility of Lassa fever infection) perceived severity (how serious is Lassa fever?)	Cross-sectional study; quantitative data; self-administered questionnaire; perceived likelihood: 4-point Likert-type scale, perceived severity: 5-point Likert-type scale
Usuwa et al. 2020 [44]	Nigeria	Lassa fever	General adult population	To investigate the knowledge and risk perception of residents towards LF and determine the factors influencing their risk perception in communities that have reported confirmed cases of LF	The health belief model	perceived susceptibility (if you do not take any preventive measures) Perceived severity (seriousness of illness in general and if contracted by respondent)	Cross-sectional study; quantitative data; interviewer-administered questionnaire; 5-point Likert-type scale
Winters et al. 2020 [68]	Sierra Leone	Ebola virus disease	General adult population	To determine how exposure to information sources, knowledge and behaviours potentially influenced risk perceptions during an Ebola Virus Disease outbreak i	KAP	Perceived likelihood (level of risk in getting Ebola in the next 6 months)	Longitudinal study (3 cross-sectional surveys, different respondents in each survey); quantitative data; interviewer-administered questionnaire; 4-point Likert-type scale
Xu et al. 2019 [55]	Myanmar	Dengue fever	General adult population	To investigate the health beliefs, knowledge and perception about dengue fever	None	Perceived likelihood (perceived risk of contracting dengue fever) Perceived severity (dengue fever is a serious illness—dengue fever is a deadly disease)	Cross-sectional study; mixed methods; interviewer-administered questionnaire, in-depth interviews; Unable to ascertain from paper
Xu et al. 2020 [52]	Myanmar	Dengue fever	General adult population	To understand health beliefs in general, and knowledge and treatment-seeking and prevention behaviours related to dengue fever	None	Perceived likelihood (perceived risk of contracting dengue fever) Perceived severity (dengue fever is a serious illness—dengue fever is a deadly disease)	Cross-sectional study; mixed methods; interviewer-administered questionnaire, in-depth interviews; unable to ascertain from paper

*KAP* knowledge, attitudes and practices

### Quality of evidence

Of the fifty-six eligible studies, we graded forty as good, twelve as acceptable and four as poor quality. The results of quality appraisal of eligible papers are presented in Additional file 2.

Among cross-sectional studies (*n* = 45), the most common weakness was not categorising, addressing or describing non-responders, or commenting on potential non-response bias. Similarly, among five qualitative studies and two mixed methods studies, none reported on the numbers or reasons of those who chose not to participate.

Among qualitative studies (*n* = 5), there was generally a lack of information on the studies’ ethical procedures, such as for informed consent or safeguarding confidentiality and anonymity. Among mixed methods studies (*n* = 6), none adequately addressed divergences and inconsistencies between qualitative and quantitative data.

### Discussion

To the best of our knowledge, this is currently the only systematic review to examine the evidence of epidemic risk perceptions in populations that are highly vulnerable to epidemics. The review highlights that, despite a moderate body of evidence, major gaps remain. Studies from only eighteen of the 62 eligible countries were identified. Diseases that cause frequent epidemics in these settings [30], such as measles or cholera, received little or no attention. This finding is similar to previous research suggesting that epidemics of common diseases are less likely to be responded to in a timely manner [84], or to be evaluated [30].

This review set out to identify how a context of frequent and often concurrent epidemics influences epidemic risk perceptions. Research on non-communicable and heritable diseases suggests that perceived risk of a disease influences the perceived risk of other diseases, and that the perceived risk does not necessarily correspond to the actual risk posed by a disease [85–87]. However, only three studies in our review compared the perception of two or more epidemic-prone diseases in the same population, and two studies explored the influence of familiarity and novelty of a disease on risk perception. Furthermore, none specifically explored the influence of the high-vulnerability context on epidemic risk perceptions. Our review highlights the need for research that explores epidemic risk perception construction in the broader context of living in a setting with frequent and multiple epidemics.

### Factors influencing epidemic risk perceptions

The review findings suggest that the general population consistently perceived their likelihood of acquiring infections as lower than they rated the severity of diseases, and they were more likely to perceive the risk of infection to others as higher than to themselves. Occupational groups with high exposure to specific diseases, such as bushmeat handlers, reported even lower perceived likelihood than the general population, and similarly perceived the risk of infection to other members of their trade as higher than to themselves. This phenomenon of lower perceived likelihood, termed ‘unrealistic optimism’ [88] and described as a cognitive bias, is often observed in the general population across cultures [89]. Optimistic bias has been found to particularly occur in a comparative assessment with risk to others [90], and during active outbreaks [91]. Our findings suggest that unrealistic optimism among some high-risk occupational groups may be explained by the long-term and well-established uses of their products and services. Epidemic responders should consider how unrealistic optimism could hinder risk communication, particularly when designing communication strategies that incorporate social comparisons of risk.

By contrast, perceived likelihood of infection was generally high amongst health professionals, though findings were inconclusive when comparing perceived risk to self with risk to colleagues. This group mainly cited concerns about their employing institutions’ ability to create a safe and effective work environment, and the effectiveness of the broader health system response, described by the SARF as the influence of the organisational response or behaviour on risk perception modification. The influence of perceived health system disaster response capacity on risk perception has been reported among health professionals in better-resourced settings, such as Singapore, Saudi Arabia and Canada [92–94]. However, factors other than organisational effectiveness remain insufficiently explored. These include the socio-cultural context and different information sources and channels, particularly in conditions of scientific uncertainty about the disease in question. Risk communication interventions to modify health professionals’ epidemic risk perceptions should therefore be accompanied with measures to enhance safety in the workplace.

Our findings suggest risk perception is influenced by disease characteristics, especially disease severity, familiarity, controllability and phase of an outbreak. Analogous associations feature at the core of Slovic’s psychometric paradigm [95] and Covello’s four theoretical risk communications models [96], to describe the psychological processes of risk perception formation. However, the SARF extends this further to explain how individuals or groups select specific characteristics of the risk, interpret them and communicate them to others, and how this selection varies across different settings and risks. Our review suggests that some information sources may be more influential than others, and that this variation may be due to different sources highlighting different disease attributes in their messages. Further research is needed into why certain disease characteristics become salient in settings with frequent epidemics, and how communication channels and content may mediate the relationship between disease characteristics and risk perception formation.

Review findings suggest that evidence on the influence of demographic factors on risk perception is inconclusive. This may indicate the diversity in conceptualisation and methods of measuring risk perception used by the studies in our review. Previous research suggests that age differences in risk may vary across the domain of risk under investigation—for example, different age groups may interpret disease ‘severity’ in terms of its health, social or economic consequences and therefore give different responses [97]. Similarly, gender differences in risk perception are reported to be sensitive to methodological approaches—for example, while women consistently demonstrate higher risk perceptions for all risks, gender differences are not observed when respondents are asked to rank hazards in order of severity or seriousness [98]. The findings suggest that risk communication interventions targeting a specific demographic should account for heterogeneous risk perceptions within that group.

The review suggests that there is insufficient evidence on how epidemic risk perceptions are formed or modified in these populations. Only a third of eligible studies in our review reported on factors influencing risk perceptions. In general, there was lack of depth to the inquiry in the studies. This may be due to most studies being cross-sectional and quantitative, precluding exploration of why people perceived what they did, and how and why risk perceptions varied between diseases, populations and over time. Studies among the public primarily focused on individual constructions of risk, such as the influence of disease attributes and socio-demographic variables, but few studies explored the role of information sources and channels, cultural factors, and none studied the influence of perceptions of the epidemic response. In contrast, studies among health professionals primarily investigated the influence of institutional efficacy on risk perception. Furthermore, the studies in our reported on the independent influence of selected factors on risk perception, but none explored the interaction between these factors to shed light on the complex process of risk perception formation or adaptation. Further research is needed to explore the differences in epidemic risk perceptions between population groups, particularly the social and cultural processes that intensify or attenuate perceptions of the disease risk and its manageability.

### Conceptualisation and measurement of epidemic risk perceptions

Our review finds that, while epidemic risk perceptions are measured in a moderate number of studies across disciplines, there is wide variation in the conceptualisation of risk perception by researchers. Overall, the review revealed limited engagement with the concept of risk perception and only a third used conceptual frameworks or models to situate their hypotheses and findings. The authors’ conceptualisations of risk perception were mostly deduced from the study variables, instruments or results. None of the studies acknowledged the effect of question wording on how respondents may rate or describe their perceived risk [99]. This is particularly relevant in settings where studies were not conducted in the English language, since the conceptualisation of risk varies widely across cultures and languages [100].

The operational definition of epidemic risk perception varied widely across studies, ranging from unidimensional or single item measures to multidimensional composite risk perception scores. Our findings indicate that most researchers measure one dimension of risk perception, usually likelihood, whereas only a minority measure a combination of dimensions, such as likelihood, severity and vulnerability. Few researchers combined measurements of probability judgements, such as likelihood and vulnerability, with consequential judgements, such as affect/feelings or severity. In their review of hazard risk perception measurement methods, Wilson et al. reported that almost half of studies measured only one dimension of risk perception, often perceived likelihood, and argued that this unidimensional approach is not particularly valid or reliable for understanding individual risk perception formation [101].

Even where different studies used the same conceptual frameworks or risk perceptions definitions, diverse measurement methods limited comparisons. It was difficult to interpret whether there were actual differences in risk perception between diseases, countries or populations, or whether observed inconsistencies were due to methodological design. For example, many eligible studies used Likert-type scales to capture risk perception responses, but the inconsistent use of ‘don’t know’ response categories by researchers complicated the interpretation of findings. Previous research indicates that a nonnegligible proportion of study respondents report not knowing their risk of diseases in studies, particularly in populations that are socio-economically disadvantaged or with health disparities [102].

While the vast majority of studies in our review were deemed of good or acceptable quality by standardised quality appraisal tools, in general, there was lack of depth to the inquiry. This may be due to the fact that the majority of studies evaluated in this review used a cross-sectional design, with most being quantitative studies, and therefore lacking in-depth and longitudinal exploration of why people perceived what they did, and if, how and why risk perceptions varied between diseases, populations and over time. Furthermore, the high level of heterogeneity in methods, tools and measurement scales in eligible studies prevented a definitive identification of factors associated with epidemic risk perceptions. Varying conceptualisations, definitions and measurements of health risk perceptions and behaviours have previously been shown to hamper cross-study comparisons [13, 20, 103].

### Review limitations

Screening and selection were conducted by a single reviewer, and may have resulted in some eligible studies being missed. To mitigate this risk, the reviewer erred on the side of caution and included items with unclear eligibility in the second stage of screening. We did not include grey literature which may have provided additional and valuable insights, particularly publications by humanitarian responders serving populations in eligible countries. Due to the heterogeneity in outcomes and study methods, only a narrative analysis and synthesis was feasible. Furthermore, it was not feasible to contextualise all of the findings from the diverse set of epidemic-prone diseases, countries and population groups included in this review; instead, we attempted to identify and describe key themes that could be useful to researchers and epidemic responders. Finally, there were limitations posed by methodological weaknesses in a minority of included studies, mainly related to non-response, ethical considerations and a lack of information on inconsistencies between qualitative and quantitative epidemic risk perception data.

## Conclusions

This review suggests that evidence on epidemic risk perception in countries at the highest risk of these public health emergencies is limited. Available studies afford some insight into patterns of epidemic risk perception and factors influencing its formation, but the quality and validity of these findings are affected by a lack of in-depth inquiry and exploration. There are several areas in particular that require more attention from researchers. First, risk perceptions of diseases that cause frequent epidemics in these settings, such as measles and cholera, should be given more attention and explored in-depth to better inform responses. Second, studies comparing perceptions of different epidemic-prone diseases in the same population, or comparing perceptions across different populations or settings are essential for better contextualisation of risk perception understanding. Third, research that adopts a comprehensive, theory-driven, and preferably longitudinal, exploration of epidemic risk perception construction is needed, particularly to situate risk perceptions in the broader context of living in a setting with frequent and multiple epidemics.

The review also suggests that the science of defining and measuring epidemic risk perception is still relatively underdeveloped. First, there is a need for promotion of best practices in measuring risk perceptions, such as the systematic inclusion of ‘don’t know’ categories in risk perception measurement scales. Such standardisation will facilitate comparisons among studies and allow for systematic accumulation of evidence. Second, more research that explores or measures multiple dimensions of epidemic risk perceptions is needed, such as studies that simultaneously explore perceived probability, vulnerability and severity.

## Supplementary Information

**Additional file 1.** Search terms, and search strategy and results by database.

**Additional file 2.** Quality appraisal of eligible studies (n = 56).

## Data Availability

All data generated or analysed during this study are included in this published article and its additional information files.

## Abbreviations

AXIS: Appraisal tool for cross-sectional studies
COVID-19: Coronavirus disease
EVD: Ebola virus disease
H1N1: Pandemic influenza A
IDP: Internally-displaced person
KAP: Knowledge, attitudes and practices
MMAT: Mixed methods appraisal tool
PPE: Personal protective equipment
SARF: Social amplification of risk framework
