# Supplementary material for: Public and health professional epidemic risk perceptions in countries that are highly vulnerable to epidemics: a systematic review
Source: Infect Dis Poverty. 2022 Jan 6;11:4. doi: 10.1186/s40249-021-00927-z (PMC8731200; doi:10.1186/s40249-021-00927-z)
Supplement: Supplementary file 1 — Additional file 1. Search terms, and search strategy and results by database. [file 40249_2021_927_MOESM1_ESM.docx]

**Additional file 1: Search terms, and search strategy and results by database**

**Search terms**

| Category | Concept | Terms for title and abstract search |
| --- | --- | --- |
| Population of interest | (62) Countries considered highly vulnerable to epidemics | [somali* or “central african*” or chad* or "south* sudan*" or marutian* or angola* or haiti* or afghan* or niger* or madagascan* or congo* or mali* or ("guinea-bissau*" or "bissau-guinean*") or benin* or gambia* or liberia* or guinea* or ("sao tome*" or "saint thomas and prince*") or "sierra leone*" or burkina* or ("comoros*" or “comorian*") or yemen* or eritrea* or togo* or mozambi* or ("congo*" or "congo-brazzaville*") or nigeria* or ("cote d'ivoire*" or "ivory coast*" or "ivorian*") or malawi* or sudan* or djibouti* or pakistan* or ("east* timor*" or "timor-leste*") or senegal* or zimbabwe* or "papua new guinea*" or tanzania* or ("lesotho*" or "basotho*" or "mosotho*") or burundi* or lao* or ("cambodia*" or "khmer*") or rwand* or ("eswatini*" or "swazi*" or "swati*") or uganda* or "solomon island*" or ("democratic people's republic of korea*" or "north korea*") or ethiopia* or kenya* or kiribati* or cameroon* or kosov* or ("marshall island*" or "marshallese*") or micronesia* or ("myanma*" or "burm*") or tuvalu* or ("palestin*" or "west bank*" or "gaza*") or ("bosnia*" or "herzegovinia*") or iraq* or leban* or libya* or syria* or venezuela*]  A subject heading search will not be conducted for this concept |
| Exposure | Epidemic-prone diseases | [Epidemic, pandemic, outbreak, epidemic-prone]  A subject heading search will also be conducted for this concept |
| Outcome | Risk perception | [risk, likelihood, susceptibility, severity, vulnerability, probability, hazard, threat, harm, loss, danger, safety, protection]    and  [perception, perceive, misperception, awareness, opinion, estimation, attitude, belief]  A subject heading search will also be conducted for this concept |

**Search strategy and results by database**

**Africa-Wide Information (28 December 2020)**

#S1 TI (risk* or likel* or susceptib* or probab* or sever* or vulnerab* or hazard* or threat* or harm* or loss* or danger* or safe* or protect*) - 130,312

#S2 TI (percept* or perceiv* or mispercept* or aware* or opinion* or estimat* or attitud* or belief*) - 44,388

#S3 AB (risk* or likel* or susceptib* or probab* or sever* or vulnerab* or hazard* or threat* or harm* or loss* or danger* or safe* or protect*) - 521,818

#S4 AB (percept* or perceiv* or mispercept* or aware* or opinion* or estimat* or attitud* or belief*) - 179,021

#S5 (s1 or s3) n2 (s2 or s4) - 10,871

#S6 TI (Epidemic* or pandemic* or outbreak* or "epidemic-prone") - 13,722

#S7 AB (Epidemic* or pandemic* or outbreak* or "epidemic-prone") - 37,146

#S8 S6 OR S7 - 44,123

#S9 TI (somali* or "central african*" or chad* or "south* sudan*" or marutian* or angola* or haiti* or afghan* or niger* or madagasca* or congo* or mali* or ("guinea-bissau*" or "bissau-guinean*") or benin* or gambia* or liberia* or guinea* or ("sao tome*" or "saint thomas and prince*") or "sierra leone*" or burkina* or ("comoros*" or "comorian*") or yemen* or eritrea* or togo* or mozambi* or ("congo*" or "congo-brazzaville*") or nigeria* or ("cote d'ivoire*" or "ivory coast*" or "ivorian*") or malawi* or sudan* or djibouti* or pakistan* or ("east* timor*" or "timor-leste*") or senegal* or zimbabwe* or "papua new guinea*" or tanzania* or ("lesotho*" or "basotho*" or "mosotho*") or burundi* or lao* or ("cambodia*" or "khmer*") or rwand* or ("eswatini*" or "swazi*" or "swati*") or uganda* or "solomon island*" or ("democratic people's republic of korea*" or "north* korea*") or ethiopia* or kenya* or kiribati* or cameroon* or kosov* or ("marshall island*" or "marshallese*") or micronesia* or ("myanma*" or "burm*") or tuvalu* or ("palestin*" or "west bank*" or "gaza*") or ("bosnia*" or "herzegovinia*") or iraq* or leban* or libya* or syria* or venezuela*) - 720,362

#S10 AB (somali* or "central african*" or chad* or "south* sudan*" or marutian* or angola* or haiti* or afghan* or niger* or madagasca* or congo* or mali* or ("guinea-bissau*" or "bissau-guinean*") or benin* or gambia* or liberia* or guinea* or ("sao tome*" or "saint thomas and prince*") or "sierra leone*" or burkina* or ("comoros*" or "comorian*") or yemen* or eritrea* or togo* or mozambi* or ("congo*" or "congo-brazzaville*") or nigeria* or ("cote d'ivoire*" or "ivory coast*" or "ivorian*") or malawi* or sudan* or djibouti* or pakistan* or ("east* timor*" or "timor-leste*") or senegal* or zimbabwe* or "papua new guinea*" or tanzania* or ("lesotho*" or "basotho*" or "mosotho*") or burundi* or lao* or ("cambodia*" or "khmer*") or rwand* or ("eswatini*" or "swazi*" or "swati*") or uganda* or "solomon island*" or ("democratic people's republic of korea*" or "north* korea*") or ethiopia* or kenya* or kiribati* or cameroon* or kosov* or ("marshall island*" or "marshallese*") or micronesia* or ("myanma*" or "burm*") or tuvalu* or ("palestin*" or "west bank*" or "gaza*") or ("bosnia*" or "herzegovinia*") or iraq* or leban* or libya* or syria* or venezuela*) - 346,716

#S11 S9 OR S10 - 874,765

#S12 S5 AND S8 AND S11 – 243

#S13 S5 AND S8 AND S11(Limiters - Published Date: 20110101-20201231; Language: English) – 153

**CINAHL Plus (28 December 2020)**

#S1 TI (risk* or likel* or susceptib* or probab* or sever* or vulnerab* or hazard* or threat* or harm* or loss* or danger* or safe* or protect*) - 499,287

#S2 AB (risk* or likel* or susceptib* or probab* or sever* or vulnerab* or hazard* or threat* or harm* or loss* or danger* or safe* or protect*) - 1,554,740

#S3 (MH "Probability+") OR (MH "Disease Susceptibility+") OR (MH "Severity of Illness+") OR (MH "Vulnerability+") OR (MH "Personal Loss+") - 201,484

#S4 TI (percept* or perceiv* or mispercept* or aware* or opinion* or estimat* or attitud* or belief*) - 148,962

#S5 AB (percept* or perceiv* or mispercept* or aware* or opinion* or estimat* or attitud* or belief*) - 572,717

#S6 (MH "Perception+") OR (MH "Perceiving (NANDA)+") - 84,904

#S7 (MH "Attitude to Risk+") OR (MH "Health Beliefs: Perceived Threat (Iowa NOC)+") OR (MH "Risk assessment+") - 111,799

#S8 (S1 OR S2 OR S3) n2 (S4 or S5 or S6) - 320,020

#S9 S7 OR S8 - 415,585

#S10 TI (Epidemic* or pandemic* or outbreak* or "epidemic-prone") - 27,843

#S11 AB (Epidemic* or pandemic* or outbreak* or "epidemic-prone") - 45,180

#S12 (MM "Disease Outbreaks+") - 24,203

#S13 S10 OR S11 OR S12 - 68,138

#S14 TI (somali* or "central african*" or chad* or "south* sudan*" or marutian* or angola* or haiti* or afghan* or niger* or madagasca* or congo* or mali* or ("guinea-bissau*" or "bissau-guinean*") or benin* or gambia* or liberia* or guinea* or ("sao tome*" or "saint thomas and prince*") or "sierra leone*" or burkina* or ("comoros*" or "comorian*") or yemen* or eritrea* or togo* or mozambi* or ("congo*" or "congo-brazzaville*") or nigeria* or ("cote d'ivoire*" or "ivory coast*" or "ivorian*") or malawi* or sudan* or djibouti* or pakistan* or ("east* timor*" or "timor-leste*") or senegal* or zimbabwe* or "papua new guinea*" or tanzania* or ("lesotho*" or "basotho*" or "mosotho*") or burundi* or lao* or ("cambodia*" or "khmer*") or rwand* or ("eswatini*" or "swazi*" or "swati*") or uganda* or "solomon island*" or ("democratic people's republic of korea*" or "north* korea*") or ethiopia* or kenya* or kiribati* or cameroon* or kosov* or ("marshall island*" or "marshallese*") or micronesia* or ("myanma*" or "burm*") or tuvalu* or ("palestin*" or "west bank*" or "gaza*") or ("bosnia*" or "herzegovinia*") or iraq* or leban* or libya* or syria* or venezuela*) - 75,174

#S15 AB (somali* or "central african*" or chad* or "south* sudan*" or marutian* or angola* or haiti* or afghan* or niger* or madagasca* or congo* or mali* or ("guinea-bissau*" or "bissau-guinean*") or benin* or gambia* or liberia* or guinea* or ("sao tome*" or "saint thomas and prince*") or "sierra leone*" or burkina* or ("comoros*" or "comorian*") or yemen* or eritrea* or togo* or mozambi* or ("congo*" or "congo-brazzaville*") or nigeria* or ("cote d'ivoire*" or "ivory coast*" or "ivorian*") or malawi* or sudan* or djibouti* or pakistan* or ("east* timor*" or "timor-leste*") or senegal* or zimbabwe* or "papua new guinea*" or tanzania* or ("lesotho*" or "basotho*" or "mosotho*") or burundi* or lao* or ("cambodia*" or "khmer*") or rwand* or ("eswatini*" or "swazi*" or "swati*") or uganda* or "solomon island*" or ("democratic people's republic of korea*" or "north* korea*") or ethiopia* or kenya* or kiribati* or cameroon* or kosov* or ("marshall island*" or "marshallese*") or micronesia* or ("myanma*" or "burm*") or tuvalu* or ("palestin*" or "west bank*" or "gaza*") or ("bosnia*" or "herzegovinia*") or iraq* or leban* or libya* or syria* or venezuela*) - 116,584

#S16 S14 OR S15 - 147,572

#S17 S9 AND S13 AND S16 – 690

#S18 S9 AND S13 AND S16 (Limiters - Published Date: 20110101-20201231; Language: English) - 549

**Embase Classic + Embase *1947 to 2020 December 24* (28 December 2020)**

1. (risk* or likel* or susceptib* or probab* or sever* or vulnerab* or hazard* or threat* or harm* or loss* or danger* or safe* or protect*).ti,ab. - 11399855

2. exp hazard/ - 736332

3. exp risk/ - 2592316

4. exp safety/ - 503919

5. exp disease severity/ - 1918179

6. 1 or 2 or 3 or 4 or 5 - 12920598

7. (percept* or perceiv* or mispercept* or aware* or opinion* or estimat* or attitud* or belief*).ti,ab. - 2717487

8. exp perception/ - 385400

9. exp awareness/ - 94648

10. exp attitude/ - 808597

11. 7 or 8 or 9 or 10 - 3505909

12. ((risk* or likel* or susceptib* or probab* or sever* or vulnerab* or hazard* or threat* or harm* or loss* or danger* or safe* or protect*).ti,ab. or exp hazard/ or exp risk/ or exp safety/ or exp disease severity/) adj2 ((percept* or perceiv* or mispercept* or aware* or opinion* or estimat* or attitud* or belief*).ti,ab. or exp perception/ or exp awareness/ or exp attitude/) - 535614

13. exp attitude to health/ - 117604

14. exp health belief/ - 11603

15. 12 or 13 or 14 - 632797

16. (epidemic* or pandemic* or outbreak* or "epidemic-prone").ti,ab. - 286461

17. exp epidemic/ - 126512

18. exp pandemic/ - 49068

19. exp pandemic influenza/ - 4871

20. 16 or 17 or 18 or 19 - 331381

21. somali*.ti,ab. - 3357

22. central africa*.ti,ab. - 5433

23. chad*.ti,ab. - 7440

24. south* sudan*.ti,ab. - 992

25. mauritan*.ti,ab. - 981

26. angola*.ti,ab. - 1851

27. haiti*.ti,ab. - 5226

28. afghan*.ti,ab. - 8180

29. niger*.ti,ab. - 64727

30. madagasca*.ti,ab. - 6199

31. congo*.ti,ab. - 20462

32. mali$3.ti,ab. - 20230

33. (guinea-bissau* or bissau-guinean*).ti,ab. - 1141

34. benin*.ti,ab. - 5603

35. gambia*.ti,ab. - 9652

36. liberia*.ti,ab. - 2122

37. guinea*.ti,ab. - 148639

38. (sao tome* or (saint thomas and prince*)).ti,ab. - 273

39. sierra leone*.ti,ab. - 2746

40. burkina*.ti,ab. - 5008

41. (comoros* or comorian*).ti,ab. - 407

42. yemen*.ti,ab. - 2897

43. eritrea*.ti,ab. - 870

44. togo*.ti,ab. - 2098

45. mozambi*.ti,ab. - 4548

46. (congo* or congo-brazaville*).ti,ab. - 20462

47. nigeria*.ti,ab. - 45939

48. (cote d'ivoire* or ivory coast* or ivorian*).ti,ab. - 4693

49. malawi*.ti,ab. - 9037

50. sudan*.ti,ab. - 13982

51. djibouti*.ti,ab. - 464

52. pakistan*.ti,ab. - 31415

53. (east timor* or timor-leste*).ti,ab. - 638

54. senegal*.ti,ab. - 9650

55. zimbabwe*.ti,ab. - 6712

56. papua new guinea*.ti,ab. - 5316

57. tanzania*.ti,ab. - 16451

58. (lesotho* or basotho* or mosotho*).ti,ab. - 831

59. burundi*.ti,ab. - 980

60. lao*.ti,ab. - 5946

61. (cambodia* or khmer*).ti,ab. - 6181

62. rwand*.ti,ab. - 4083

63. (eswatini* or swazi* or swati*).ti,ab. - 1170

64. uganda*.ti,ab. - 20070

65. solomon islan*.ti,ab. - 984

66. (democratic people's republic of korea* or north* korea*).ti,ab. - 679

67. ethiopia*.ti,ab. - 20616

68. kenya*.ti,ab. - 25075

69. kiribati*.ti,ab. - 203

70. cameroon*.ti,ab. - 9051

71. kosov*.ti,ab. - 1558

72. (marshall island* or marshallese*).ti,ab. - 492

73. micronesia*.ti,ab. - 1020

74. (myanma* or burm*).ti,ab. - 10001

75. tuvalu*.ti,ab. - 74

76. (palestin* or west bank* or gaza*).ti,ab. - 4775

77. (bosnia* or herzegovinia*).ti,ab. - 3956

78. iraq*.ti,ab. - 14582

79. leban*.ti,ab. - 7908

80. libya*.ti,ab. - 2226

81. syria*.ti,ab. - 15141

82. venezuela*.ti,ab. - 10486

83. 21 or 22 or 23 or 24 or 25 or 26 or 27 or 28 or 29 or 30 or 31 or 32 or 33 or 34 or 35 or 36 or 37 or 38 or 39 or 40 or 41 or 42 or 43 or 44 or 45 or 46 or 47 or 48 or 49 or 50 or 51 or 52 or 53 or 54 or 55 or 56 or 57 or 58 or 59 or 60 or 61 or 62 or 63 or 64 or 65 or 66 or 67 or 68 or 69 or 70 or 71 or 72 or 73 or 74 or 75 or 76 or 77 or 78 or 79 or 80 or 81 or 82 - 538407

84. 15 and 20 and 83 - 989

85. limit 84 to yr="2011 - 2020" - 738

86. limit 85 to english language - 726

**Global Health *1910 to 2020 Week 51* (28 December 2020)**

1. (risk* or likel* or susceptib* or probab* or sever* or vulnerab* or hazard* or threat* or harm* or loss* or danger* or safe* or protect*).ti,ab. - 1671835

2. exp hazards/ - 9631

3. exp risk/ - 77228

4. exp susceptibility/ - 64930

5. exp probability/ - 360

6. exp health hazards/ - 75011

7. exp safety/ - 139717

8. exp protection/ - 34243

9. 1 or 2 or 3 or 4 or 5 or 6 or 7 or 8 - 1736632

10. (percept* or perceiv* or mispercept* or aware* or opinion* or estimat* or attitud* or belief*).ti,ab. - 512812

11. exp perception/ - 4388

12. exp awareness/ - 7935

13. exp attitudes/ - 66006

14. exp opinions/ - 3892

15. exp beliefs/ - 13952

16. 10 or 11 or 12 or 13 or 14 or 15 - 529923

17. ((risk* or likel* or susceptib* or probab* or sever* or vulnerab* or hazard* or threat* or harm* or loss* or danger* or safe* or protect*).ti,ab. or exp hazards/ or exp risk/ or exp susceptibility/ or exp probability/ or exp health hazards/ or exp safety/ or exp protection/) adj2 ((percept* or perceiv* or mispercept* or aware* or opinion* or estimat* or attitud* or belief*).ti,ab. or exp perception/ or exp awareness/ or exp attitudes/ or exp opinions/ or exp beliefs/) - 49038

18. exp risk assessment/ - 62853

19. 17 or 18 - 106703

20. (epidemic* or pandemic* or outbreak* or "epidemic-prone").ti,ab. - 154332

21. exp epidemics/ - 47551

22. exp pandemics/ - 9846

23. exp outbreaks/ - 56008

24. 20 or 21 or 22 or 23 - 157026

25. somali*.ti,ab. - 2515

26. central africa*.ti,ab. - 3922

27. chad*.ti,ab. - 1662

28. south* sudan*.ti,ab. - 934

29. mauritan*.ti,ab. - 811

30. angola*.ti,ab. - 1667

31. haiti*.ti,ab. - 2679

32. afghan*.ti,ab. - 2632

33. niger*.ti,ab. - 45834

34. madagasca*.ti,ab. - 4337

35. congo*.ti,ab. - 12768

36. mali$3.ti,ab. - 6759

37. (guinea-bissau* or bissau-guinean*).ti,ab. - 833

38. benin*.ti,ab. - 3625

39. gambia*.ti,ab. - 11191

40. liberia*.ti,ab. - 1997

41. guinea*.ti,ab. - 42830

42. (sao tome* or (saint thomas and prince*)).ti,ab. - 198

43. sierra leone*.ti,ab. - 2355

44. burkina*.ti,ab. - 4102

45. (comoros* or comorian*).ti,ab. - 291

46. yemen*.ti,ab. - 1856

47. eritrea*.ti,ab. - 764

48. togo*.ti,ab. - 2362

49. mozambi*.ti,ab. - 3298

50. (congo* or congo-brazaville*).ti,ab. - 12768

51. nigeria*.ti,ab. - 34093

52. (cote d'ivoire* or ivory coast* or ivorian*).ti,ab. - 4816

53. malawi*.ti,ab. - 6099

54. sudan*.ti,ab. - 9456

55. djibouti*.ti,ab. - 352

56. pakistan*.ti,ab. - 15586

57. (east timor* or timor-leste*).ti,ab. - 353

58. senegal*.ti,ab. - 7157

59. zimbabwe*.ti,ab. - 4482

60. papua new guinea*.ti,ab. - 3572

61. tanzania*.ti,ab. - 11776

62. (lesotho* or basotho* or mosotho*).ti,ab. - 604

63. burundi*.ti,ab. - 811

64. lao*.ti,ab. - 2622

65. (cambodia* or khmer*).ti,ab. - 3580

66. rwand*.ti,ab. - 2347

67. (eswatini* or swazi* or swati*).ti,ab. - 920

68. uganda*.ti,ab. - 14350

69. solomon islan*.ti,ab. - 841

70. (democratic people's republic of korea* or north* korea*).ti,ab. - 284

71. ethiopia*.ti,ab. - 15323

72. kenya*.ti,ab. - 18533

73. kiribati*.ti,ab. - 124

74. cameroon*.ti,ab. - 7415

75. kosov*.ti,ab. - 589

76. (marshall island* or marshallese*).ti,ab. - 203

77. micronesia*.ti,ab. - 457

78. (myanma* or burm*).ti,ab. - 6763

79. tuvalu*.ti,ab. - 53

80. (palestin* or west bank* or gaza*).ti,ab. - 2599

81. (bosnia* or herzegovinia*).ti,ab. - 1297

82. iraq*.ti,ab.- 4564

83. leban*.ti,ab. - 2740

84. libya*.ti,ab. - 1216

85. syria*.ti,ab. - 3719

86. venezuela*.ti,ab. - 8505

87. 25 or 26 or 27 or 28 or 29 or 30 or 31 or 32 or 33 or 34 or 35 or 36 or 37 or 38 or 39 or 40 or 41 or 42 or 43 or 44 or 45 or 46 or 47 or 48 or 49 or 50 or 51 or 52 or 53 or 54 or 55 or 56 or 57 or 58 or 59 or 60 or 61 or 62 or 63 or 64 or 65 or 66 or 67 or 68 or 69 or 70 or 71 or 72 or 73 or 74 or 75 or 76 or 77 or 78 or 79 or 80 or 81 or 82 or 83 or 84 or 85 or 86 - 274685

88. 19 and 24 and 87 - 505

89. limit 88 to yr="2011 - 2020" - 398

90. limit 89 to english language - 365

**Ovid MEDLINE® *1946 to December Week 3 2020* (28 December 2020)**

1. (risk* or likel* or susceptib* or probab* or sever* or vulnerab* or hazard* or threat* or harm* or loss* or danger* or safe* or protect*).ti,ab. - 6994372

2. exp risk/ - 1236857

3. exp disease susceptibility/ - 171043

4. exp safety/ - 82162

5. 1 or 2 or 3 or 4 - 7362907

6. (percept* or perceiv* or mispercept* or aware* or opinion* or estimat* or attitud* or belief*).ti,ab. - 1674050

7. exp perception/ - 436071

8. exp awareness/ - 20479

9. exp attitude/ - 584105

10. 6 or 7 or 8 or 9 - 2349079

11. ((risk* or likel* or susceptib* or probab* or sever* or vulnerab* or hazard* or threat* or harm* or loss* or danger* or safe* or protect*).ti,ab. or exp risk/ or exp disease susceptibility/ or exp safety/) adj2 ((percept* or perceiv* or mispercept* or aware* or opinion* or estimat* or attitud* or belief*).ti,ab. or exp perception/ or exp awareness/ or exp attitude/) - 326362

12. exp risk assessment/ - 277184

13. 11 or 12 - 576140

14. (epidemic* or pandemic* or outbreak* or "epidemic-prone").ti,ab. - 195666

15. exp epidemics/ - 56872

16. exp pandemics/ - 45984

17. exp disease outbreaks/ - 137403

18. 14 or 15 or 16 or 17 - 244325

19. somali*.ti,ab. - 2395

20. central africa*.ti,ab. - 4161

21. chad*.ti,ab. - 2905

22. south* sudan*.ti,ab. - 729

23. mauritan*.ti,ab. - 758

24. angola*.ti,ab. - 1399

25. haiti*.ti,ab. - 3580

26. afghan*.ti,ab. - 5731

27. niger*.ti,ab. - 40645

28. madagasca*.ti,ab. - 4688

29. congo*.ti,ab. - 13180

30. mali$3.ti,ab. - 12353

31. (guinea-bissau* or bissau-guinean*).ti,ab. - 899

32. benin*.ti,ab. - 3173

33. gambia*.ti,ab. - 7439

34. liberia*.ti,ab. - 1526

35. guinea*.ti,ab. - 103736

36. (sao tome* or (saint thomas and prince*)).ti,ab. - 235

37. sierra leone*.ti,ab. - 1893

38. burkina*.ti,ab. - 3544

39. (comoros* or comorian*).ti,ab. - 328

40. yemen*.ti,ab. - 1920

41. eritrea*.ti,ab. - 584

42. togo*.ti,ab. - 1565

43. mozambi*.ti,ab.- 3278

44. (congo* or congo-brazaville*).ti,ab. - 13180

45. nigeria*.ti,ab. - 28573

46. (cote d'ivoire* or ivory coast* or ivorian*).ti,ab. - 3527

47. malawi*.ti,ab. - 6389

48. sudan*.ti,ab. - 8465

49. djibouti*.ti,ab. - 357

50. pakistan*.ti,ab. - 17578

51. (east timor* or timor-leste*).ti,ab. - 453

52. senegal*.ti,ab. - 6930

53. zimbabwe*.ti,ab. - 5372

54. papua new guinea*.ti,ab. - 4393

55. tanzania*.ti,ab. - 11721

56. (lesotho* or basotho* or mosotho*).ti,ab. - 621

57. burundi*.ti,ab. - 741

58. lao*.ti,ab. - 3971

59. (cambodia* or khmer*).ti,ab. - 4322

60. rwand*.ti,ab. - 2727

61. (eswatini* or swazi* or swati*).ti,ab. - 832

62. uganda*.ti,ab. - 13333

63. solomon islan*.ti,ab. - 755

64. (democratic people's republic of korea* or north* korea*).ti,ab. - 438

65. ethiopia*.ti,ab. - 14087

66. kenya*.ti,ab. - 17655

67. kiribati*.ti,ab. - 168

68. cameroon*.ti,ab. - 6451

69. kosov*.ti,ab. - 894

70. (marshall island* or marshallese*).ti,ab. - 343

71. micronesia*.ti,ab. - 796

72. (myanma* or burm*).ti,ab. - 6358

73. tuvalu*.ti,ab. - 60

74. (palestin* or west bank* or gaza*).ti,ab. - 3121

75. (bosnia* or herzegovinia*).ti,ab. - 2470

76. iraq*.ti,ab. - 7424

77. leban*.ti,ab. - 4896

78. libya*.ti,ab. - 1384

79. syria*.ti,ab. - 11194

80. venezuela*.ti,ab. - 7500

81. 19 or 20 or 21 or 22 or 23 or 24 or 25 or 26 or 27 or 28 or 29 or 30 or 31 or 32 or 33 or 34 or 35 or 36 or 37 or 38 or 39 or 40 or 41 or 42 or 43 or 44 or 45 or 46 or 47 or 48 or 49 or 50 or 51 or 52 or 53 or 54 or 55 or 56 or 57 or 58 or 59 or 60 or 61 or 62 or 63 or 64 or 65 or 66 or 67 or 68 or 69 or 70 or 71 or 72 or 73 or 74 or 75 or 76 or 77 or 78 or 79 or 80 - 358786

82. 13 and 18 and 81 - 666

83. limit 82 to yr="2011 - 2020" - 418

84. limit 83 to english language – 409

**APA PsycINFO** ***1806 to December Week 3 2020* (28 December 2020)**

1. (risk* or likel* or susceptib* or probab* or sever* or vulnerab* or hazard* or threat* or harm* or loss* or danger* or safe* or protect*).ti,ab. - 1447366

2. exp hazards/ - 25638

3. exp mortality risk/ - 636

4. exp "susceptibility (disorders)"/ - 45676

5. exp "severity (disorders)"/ - 18537

6. exp threat/ - 10892

7. exp dangerousness/ - 1411

8. exp safety/ - 28480

9. 1 or 2 or 3 or 4 or 5 or 6 or 7 or 8 - 1465376

10. (percept* or perceiv* or mispercept* or aware* or opinion* or estimat* or attitud* or belief*).ti,ab. - 1036722

11. exp perception/ - 428344

12. exp estimation/ - 15931

13. exp attitudes/ - 390165

14. 10 or 11 or 12 or 13 - 1442052

15. ((risk* or likel* or susceptib* or probab* or sever* or vulnerab* or hazard* or threat* or harm* or loss* or danger* or safe* or protect*).ti,ab. or exp hazards/ or exp mortality risk/ or exp "susceptibility (disorders)"/ or exp "severity (disorders)"/ or exp threat/ or exp dangerousness/ or exp safety/) adj2 ((percept* or perceiv* or mispercept* or aware* or opinion* or estimat* or attitud* or belief*).ti,ab. or exp perception/ or exp estimation/ or exp attitudes/) - 141618

16. exp risk perception/ - 6882

17. exp risk assessment/ - 13819

18. exp probability judgment/ - 1816

19. exp threat assessment/ - 202

20. exp health awareness/ - 298

21. 15 or 16 or 17 or 18 or 19 or 20 - 155840

22. (epidemic* or pandemic* or outbreak* or "epidemic-prone").ti,ab. - 18638

23. exp disease outbreaks/ - 4630

24. exp epidemics/ - 4582

25. exp pandemics/ - 1502

26. 22 or 23 or 24 or 25 - 19001

27. somali*.ti,ab. - 923

28. central africa*.ti,ab. - 319

29. chad*.ti,ab. - 439

30. south* sudan*.ti,ab. - 193

31. mauritan*.ti,ab. - 51

32. angola*.ti,ab. - 212

33. haiti*.ti,ab. - 1364

34. afghan*.ti,ab. - 3218

35. niger*.ti,ab. - 6332

36. madagasca*.ti,ab. – 568

37. congo*.ti,ab. – 989

38. mali$3.ti,ab. - 1052

39. (guinea-bissau* or bissau-guinean*).ti,ab. - 67

40. benin*.ti,ab. - 296

41. gambia*.ti,ab. - 247

42. liberia*.ti,ab. - 445

43. guinea*.ti,ab. - 4273

44. (sao tome* or (saint thomas and prince*)).ti,ab. - 20

45. sierra leone*.ti,ab. - 486

46. burkina*.ti,ab. - 332

47. (comoros* or comorian*).ti,ab. - 14

48. yemen*.ti,ab. - 296

49. eritrea*.ti,ab. - 153

50. togo*.ti,ab. - 161

51. mozambi*.ti,ab. - 520

52. (congo* or congo-brazaville*).ti,ab. - 989

53. nigeria*.ti,ab. - 6039

54. (cote d'ivoire* or ivory coast* or ivorian*).ti,ab. - 408

55. malawi*.ti,ab. - 1118

56. sudan*.ti,ab. - 814

57. djibouti*.ti,ab. - 23

58. pakistan*.ti,ab. - 3694

59. (east timor* or timor-leste*).ti,ab. - 152

60. senegal*.ti,ab. - 746

61. zimbabwe*.ti,ab. - 1334

62. papua new guinea*.ti,ab. - 635

63. tanzania*.ti,ab. - 2100

64. (lesotho* or basotho* or mosotho*).ti,ab. - 227

65. burundi*.ti,ab. - 174

66. lao*.ti,ab. - 726

67. (cambodia* or khmer*).ti,ab. - 1386

68. rwand*.ti,ab. - 1016

69. (eswatini* or swazi* or swati*).ti,ab. - 278

70. uganda*.ti,ab. - 3045

71. solomon islan*.ti,ab. - 126

72. (democratic people's republic of korea* or north* korea*).ti,ab. - 243

73. ethiopia*.ti,ab. - 2063

74. kenya*.ti,ab. - 3673

75. kiribati*.ti,ab. - 23

76. cameroon*.ti,ab. - 685

77. kosov*.ti,ab. - 423

78. (marshall island* or marshallese*).ti,ab. - 81

79. micronesia*.ti,ab. - 195

80. (myanma* or burm*).ti,ab. - 800

81. tuvalu*.ti,ab. - 19

82. (palestin* or west bank* or gaza*).ti,ab. - 3111

83. (bosnia* or herzegovinia*).ti,ab. - 1058

84. iraq*.ti,ab. - 5171

85. leban*.ti,ab. - 1948

86. libya*.ti,ab. - 235

87. syria*.ti,ab. - 1758

88. venezuela*.ti,ab. - 838

89. 27 or 28 or 29 or 30 or 31 or 32 or 33 or 34 or 35 or 36 or 37 or 38 or 39 or 40 or 41 or 42 or 43 or 44 or 45 or 46 or 47 or 48 or 49 or 50 or 51 or 52 or 53 or 54 or 55 or 56 or 57 or 58 or 59 or 60 or 61 or 62 or 63 or 64 or 65 or 66 or 67 or 68 or 69 or 70 or 71 or 72 or 73 or 74 or 75 or 76 or 77 or 78 or 79 or 80 or 81 or 82 or 83 or 84 or 85 or 86 or 87 or 88 - 54406

90. 21 and 26 and 89 - 114

91. limit 90 to yr="2011 - 2020" - 60

92. limit 91 to english language – 60

**Web of Science Core Collection (28 December 2020)**

#1 TS=((risk* near/2 percept*) or (risk* near/2 perceiv*) or (risk* near/2 mispercept*) or (risk* near/2 aware*) or (risk* near/2 opinion*) or (risk* near/2 estimat*) or (risk* near/2 attitud*) or (risk* near/2 belief*) )

*Indexes=SCI-EXPANDED, SSCI, A&HCI, CPCI-S, CPCI-SSH, ESCI Timespan=All years*

- 96054

#2 TS=((likel* near/2 percept*) or (likel* near/2 perceiv*) or (likel* near/2 mispercept*) or (likel* near/2 aware*) or (likel* near/2 opinion*) or (likel* near/2 estimat*) or (likel* near/2 attitud*) or (likel* near/2 belief*) )

*Indexes=SCI-EXPANDED, SSCI, A&HCI, CPCI-S, CPCI-SSH, ESCI Timespan=All years*

- 56045

#3 TS=((susceptib* near/2 percept*) or (susceptib* near/2 perceiv*) or (susceptib* near/2 mispercept*) or (susceptib* near/2 aware*) or (susceptib* near/2 opinion*) or (susceptib* near/2 estimat*) or (susceptib* near/2 attitud*) or (susceptib* near/2 belief*) )

*Indexes=SCI-EXPANDED, SSCI, A&HCI, CPCI-S, CPCI-SSH, ESCI Timespan=All years*

- 3213

#4 TS=((sever* near/2 percept*) or (sever* near/2 perceiv*) or (sever* near/2 mispercept*) or (sever* near/2 aware*) or (sever* near/2 opinion*) or (sever* near/2 estimat*) or (sever* near/2 attitud*) or (sever* near/2 belief*) )

*Indexes=SCI-EXPANDED, SSCI, A&HCI, CPCI-S, CPCI-SSH, ESCI Timespan=All years*

- 22559

#5 TS=((vulnerab* near/2 percept*) or (vulnerab* near/2 perceiv*) or (vulnerab* near/2 mispercept*) or (vulnerab* near/2 aware*) or (vulnerab* near/2 opinion*) or (vulnerab* near/2 estimat*) or (vulnerab* near/2 attitud*) or (vulnerab* near/2 belief*) )

*Indexes=SCI-EXPANDED, SSCI, A&HCI, CPCI-S, CPCI-SSH, ESCI Timespan=All years*

- 3298

#6 TS=((probab* near/2 percept*) or (probab* near/2 perceiv*) or (probab* near/2 mispercept*) or (probab* near/2 aware*) or (probab* near/2 opinion*) or (probab* near/2 estimat*) or (probab* near/2 attitud*) or (probab* near/2 belief*) )

*Indexes=SCI-EXPANDED, SSCI, A&HCI, CPCI-S, CPCI-SSH, ESCI Timespan=All years*

- 37452

#7 TS=((hazard* near/2 percept*) or (hazard* near/2 perceiv*) or (hazard* near/2 mispercept*) or (hazard* near/2 aware*) or (hazard* near/2 opinion*) or (hazard* near/2 estimat*) or (hazard* near/2 attitud*) or (hazard* near/2 belief*) )

*Indexes=SCI-EXPANDED, SSCI, A&HCI, CPCI-S, CPCI-SSH, ESCI Timespan=All years*

- 12332

#8 TS=((threat* near/2 percept*) or (threat* near/2 perceiv*) or (threat* near/2 mispercept*) or (threat* near/2 aware*) or (threat* near/2 opinion*) or (threat* near/2 estimat*) or (threat* near/2 attitud*) or (threat* near/2 belief*) )

*Indexes=SCI-EXPANDED, SSCI, A&HCI, CPCI-S, CPCI-SSH, ESCI Timespan=All years*

- 8147

#9 TS=((harm* near/2 percept*) or (harm* near/2 perceiv*) or (harm* near/2 mispercept*) or (harm* near/2 aware*) or (harm* near/2 opinion*) or (harm* near/2 estimat*) or (harm* near/2 attitud*) or (harm* near/2 belief*) )

*Indexes=SCI-EXPANDED, SSCI, A&HCI, CPCI-S, CPCI-SSH, ESCI Timespan=All years*

- 5747

#10 TS=((loss* near/2 percept*) or (loss* near/2 perceiv*) or (loss* near/2 mispercept*) or (loss* near/2 aware*) or (loss* near/2 opinion*) or (loss* near/2 estimat*) or (loss* near/2 attitud*) or (loss* near/2 belief*) )

*Indexes=SCI-EXPANDED, SSCI, A&HCI, CPCI-S, CPCI-SSH, ESCI Timespan=All years*

- 26765

#11 S=((danger* near/2 percept*) or (danger* near/2 perceiv*) or (danger* near/2 mispercept*) or (danger* near/2 aware*) or (danger* near/2 opinion*) or (danger* near/2 estimat*) or (danger* near/2 attitud*) or (danger* near/2 belief*) )

*Indexes=SCI-EXPANDED, SSCI, A&HCI, CPCI-S, CPCI-SSH, ESCI Timespan=All years*

- 2523

#12 TS=((safe* near/2 percept*) or (safe* near/2 perceiv*) or (safe* near/2 mispercept*) or (safe* near/2 aware*) or (safe* near/2 opinion*) or (safe* near/2 estimat*) or (safe* near/2 attitud*) or (safe* near/2 belief*) )

*Indexes=SCI-EXPANDED, SSCI, A&HCI, CPCI-S, CPCI-SSH, ESCI Timespan=All years*

- 13404

#13 TS=((protect* near/2 percept*) or (protect* near/2 perceiv*) or (protect* near/2 mispercept*) or (protect* near/2 aware*) or (protect* near/2 opinion*) or (protect* near/2 estimat*) or (protect* near/2 attitud*) or (protect* near/2 belief*) )

*Indexes=SCI-EXPANDED, SSCI, A&HCI, CPCI-S, CPCI-SSH, ESCI Timespan=All years*

- 4929

#14 TS= (epidemic* or pandemic* or outbreak* or epidemic-prone)

*Indexes=SCI-EXPANDED, SSCI, A&HCI, CPCI-S, CPCI-SSH, ESCI Timespan=All years*

- 282619

#15 TS = (somali* or "central african*" or chad* or "south* sudan*" or marutian* or angola* or haiti* or afghan* or niger* or madagascan* or congo* or mali* or ("guinea-bissau*" or "bissau-guinean*") or benin* or gambia* or liberia* or guinea* or ("sao tome*" or "saint thomas and prince*") or "sierra leone*" or burkina* or ("comoros*" or “comorian*") or yemen* or eritrea* or togo* or mozambi* or ("congo*" or "congo-brazzaville*") or nigeria* or ("cote d'ivoire*" or "ivory coast*" or "ivorian*") or malawi* or sudan* or djibouti* or pakistan* or ("east* timor*" or "timor-leste*") or senegal* or zimbabwe* or "papua new guinea*" or tanzania* or ("lesotho*" or "basotho*" or "mosotho*") or burundi* or lao* or ("cambodia*" or "khmer*") or rwand* or ("eswatini*" or "swazi*" or "swati*") or uganda* or "solomon island*" or ("democratic people's republic of korea*" or "north korea*") or ethiopia* or kenya* or kiribati* or cameroon* or kosov* or ("marshall island*" or "marshallese*") or micronesia* or ("myanma*" or "burm*") or tuvalu* or ("palestin*" or "west bank*" or "gaza*") or ("bosnia*" or "herzegovinia*") or iraq* or leban* or libya* or syria* or venezuela*)

*Indexes=SCI-EXPANDED, SSCI, A&HCI, CPCI-S, CPCI-SSH, ESCI Timespan=All years*

- 1398946

#16 #15 AND #14 AND #1

*Indexes=SCI-EXPANDED, SSCI, A&HCI, CPCI-S, CPCI-SSH, ESCI Timespan=All years*

- 252

#17 #15 AND #14 AND #2

*Indexes=SCI-EXPANDED, SSCI, A&HCI, CPCI-S, CPCI-SSH, ESCI Timespan=All years*

- 38

#18 #15 AND #14 AND #3

*Indexes=SCI-EXPANDED, SSCI, A&HCI, CPCI-S, CPCI-SSH, ESCI Timespan=All years*

- 12

#19 #15 AND #14 AND #4

*Indexes=SCI-EXPANDED, SSCI, A&HCI, CPCI-S, CPCI-SSH, ESCI Timespan=All years*

- 26

#20 #15 AND #14 AND #5

*Indexes=SCI-EXPANDED, SSCI, A&HCI, CPCI-S, CPCI-SSH, ESCI Timespan=All years*

- 8

#21 #15 AND #14 AND #6

*Indexes=SCI-EXPANDED, SSCI, A&HCI, CPCI-S, CPCI-SSH, ESCI Timespan=All years*

- 37

#22 #15 AND #14 AND #7

*Indexes=SCI-EXPANDED, SSCI, A&HCI, CPCI-S, CPCI-SSH, ESCI Timespan=All years*

- 11

#23 #15 AND #14 AND #8

*Indexes=SCI-EXPANDED, SSCI, A&HCI, CPCI-S, CPCI-SSH, ESCI Timespan=All years*

- 28

#24 #15 AND #14 AND #9

*Indexes=SCI-EXPANDED, SSCI, A&HCI, CPCI-S, CPCI-SSH, ESCI Timespan=All years*

- 7

#25 #15 AND #14 AND #10

*Indexes=SCI-EXPANDED, SSCI, A&HCI, CPCI-S, CPCI-SSH, ESCI Timespan=All years*

- 25

#26 #15 AND #14 AND #11

*Indexes=SCI-EXPANDED, SSCI, A&HCI, CPCI-S, CPCI-SSH, ESCI Timespan=All years*

- 4

#27 #15 AND #14 AND #12

*Indexes=SCI-EXPANDED, SSCI, A&HCI, CPCI-S, CPCI-SSH, ESCI Timespan=All years*

- 16

#28 #15 AND #14 AND #13

*Indexes=SCI-EXPANDED, SSCI, A&HCI, CPCI-S, CPCI-SSH, ESCI Timespan=All years*

- 17

#29 #28 OR #27 OR #26 OR #25 OR #24 OR #23 OR #22 OR #21 OR #20 OR #19 OR #18 OR #17 OR #16

*Indexes=SCI-EXPANDED, SSCI, A&HCI, CPCI-S, CPCI-SSH, ESCI Timespan=All years*

- 438

#30 #28 OR #27 OR #26 OR #25 OR #24 OR #23 OR #22 OR #21 OR #20 OR #19 OR #18 OR #17 OR #16

*Indexes=SCI-EXPANDED, SSCI, A&HCI, CPCI-S, CPCI-SSH, ESCI Timespan=2011-2020*

- 320
