# Supplementary material for: Public and health professional epidemic risk perceptions in countries that are highly vulnerable to epidemics: a systematic review
Source: Infect Dis Poverty. 2022 Jan 6;11:4. doi: 10.1186/s40249-021-00927-z (PMC8731200; doi:10.1186/s40249-021-00927-z)
Supplement: Supplementary file 2 — Additional file 2. Quality appraisal of eligible studies (n = 56). [file 40249_2021_927_MOESM2_ESM.docx]

**Additional file 2: Quality appraisal of eligible studies (n=56)**

**Appraisal tool for Cross-Sectional Studies (AXIS) – n=45**

| **Author(s) and year** | **Introduction** | **Methods** | | | | | | | | | | **Results** | | | | | **Discussion** | | **Other** | |
| --- | --- | --- | --- | --- | --- | --- | --- | --- | --- | --- | --- | --- | --- | --- | --- | --- | --- | --- | --- | --- |
|  | Were the aims/objectives of the study clear? | Was the study design appropriate for the state aim(s)? | Was the sample size justified? | Was the target/reference population clearly defined? (is it clear who the research was about?) | Was the sample frame taken from an appropriate population base so that it closely represented the target/reference population under investigation? | Was the selection process likely to select subjects/participants that were representative of the target/reference population under investigation? | Were measures undertaken to address and categorise non-responders? | Were the risk factor and outcome variables measured appropriate to the aims of the study? | Were the risk factor and outcome variables measured correctly using instruments/measurement that had been trialled, piloted or published previously? | Is it clear what was used to determine statistical significance and/or precision estimates? (e.g. p-values, confidence intervals) | Were the methods (including statistical methods) sufficiently described to enable them to be repeated? | Were the basic data adequately described? | Does the response rate raise concerns about non-response bias? | If appropriate, was information about non-responders described? | Were the results internally consistent? | Were the results presented for all the analyses described in the methods? | Were the authors' discussions and conclusions justified by the results? | Were the limitations of the study discussed? | Were there any funding sources or conflicts of interest that may affect the authors' interpretation of the results? | Was ethical approval or consent of participants attained? |
| Abdi, Affognon et al. 2015 | Y | Y | Y | Y | Y | Y | Y | Y | Y | N | Y | Y | N | N | Y | Y | Y | N | N | Y |
| Abou-Abbas, Nasser et al. 2020 | Y | Y | Y | Y | Y | Y | DK | Y | Y | Y | Y | Y | Y | N | Y | Y | Y | Y | N | DK |
| Adhena and Hidru 2020 | Y | Y | Y | Y | Y | Y | DK | Y | Y | Y | Y | Y | N | N | Y | Y | Y | Y | N | Y |
| Akalu, Ayelign et al. 2020 | Y | Y | Y | Y | Y | Y | DK | Y | Y | Y | Y | Y | DK | N | Y | Y | Y | Y | N | Y |
| Akram, Khan et al. 2015 | Y | Y | N | N | DK | DK | DK | Y | N | N | N | Y | DK | N | Y | Y | Y | N | N | Y |
| Alyousefi, Abdul-Ghani et al. 2016 | Y | Y | Y | Y | Y | Y | DK | Y | N | Y | Y | Y | N | N | Y | Y | Y | Y | N | Y |
| Asnakew, Asrese et al. 2020 | Y | Y | N | N | DK | DK | DK | Y | Y | N | N | Y | DK | Y | Y | Y | Y | Y | N | Y |
| Berman, Figueroa et al. 2017 | Y | Y | Y | Y | Y | Y | DK | Y | DK | N | N | Y | N | N | Y | Y | Y | Y | DK | N |
| Chaudhary, Ahmad et al. 2020 | Y | Y | N | Y | DK | Y | N | Y | Y | Y | Y | Y | DK | N | Y | Y | Y | Y | N | Y |
| Coulibaly, Nzussouo et al. 2013 | Y | Y | Y | Y | Y | Y | N | Y | N | Y | Y | Y | N | N | Y | Y | Y | Y | N | Y |
| Echoru, Kasozi et al. 2020 | Y | Y | Y | Y | Y | DK | N | Y | Y | Y | Y | Y | DK | N | Y | Y | Y | Y | N | Y |
| Ekra, Cherif et al. 2017 | Y | Y | Y | Y | Y | Y | DK | Y | Y | Y | Y | Y | N | Y | Y | Y | Y | Y | N | Y |
| Ernst, Hayden et al. 2016 | Y | Y | Y | Y | Y | Y | Y | Y | DK | N | Y | Y | DK | N | Y | Y | Y | Y | N | Y |
| Fatiregun, Adeyemo et al. 2012 | Y | Y | Y | Y | Y | Y | Y | Y | DK | Y | Y | Y | DK | N | Y | Y | Y | Y | N | Y |
| Ghazi, Taher et al. 2020 | Y | Y | Y | Y | DK | DK | N | Y | Y | N | N | Y | DK | N | Y | DK | Y | Y | N | Y |
| Gidado, Oladimeji et al. 2015 | Y | Y | Y | Y | Y | Y | N | Y | Y | Y | Y | Y | DK | N | Y | Y | Y | N | N | Y |
| Girma, Agenagnew et al. 2020 | Y | Y | N | Y | DK | DK | N | Y | Y | N | N | Y | DK | N | Y | DK | Y | Y | N | Y |
| Girum, Hailemikael et al. 2017 | Y | Y | Y | Y | Y | Y | N | Y | Y | Y | Y | Y | N | Y | Y | Y | Y | Y | N | Y |
| Hakim, Khattak et al. 2020 | Y | Y | N | Y | DK | DK | N | Y | Y | Y | Y | Y | DK | N | Y | Y | Y | Y | DK | Y |
| Idris, Inem et al. 2015 | Y | Y | N | Y | DK | Y | DK | Y | DK | Y | Y | Y | DK | N | Y | Y | Y | Y | N | Y |
| Ilesanmi and Afolabi 2020 | Y | Y | Y | Y | DK | Y | N | Y | Y | Y | Y | Y | DK | N | Y | Y | Y | Y | N | N |
| Iliyasu, Ogoina et al. 2015 | Y | Y | N | N | DK | N | N | Y | Y | Y | Y | Y | DK | N | Y | Y | Y | Y | N | Y |
| Iorfa, Ottu et al. 2020 | Y | Y | N | Y | DK | Y | N | Y | Y | N | Y | Y | DK | N | Y | Y | Y | Y | DK | Y |
| Irwin, Jalloh et al. 2017 | Y | Y | Y | Y | Y | Y | N | Y | Y | Y | Y | Y | N | Y | Y | Y | Y | Y | N | Y |
| Jalloh, Li et al. 2018 | Y | Y | Y | Y | Y | Y | N | Y | Y | Y | Y | Y | N | N | Y | Y | Y | Y | N | Y |
| Jiang, Shi et al. 2016 | Y | Y | N | Y | Y | Y | N | Y | DK | N | N | Y | DK | N | Y | Y | Y | Y | N | Y |
| Kabito, Alemayehu et al. 2020 | Y | Y | Y | Y | Y | Y | N | Y | Y | Y | Y | Y | N | N | Y | Y | Y | Y | N | Y |
| Kaponda, Muthukrishnan et al. 2019 | Y | Y | Y | Y | Y | Y | N | Y | Y | Y | Y | N | DK | N | Y | Y | Y | Y | DK | Y |
| Kasereka, Sawatzky et al. 2019 | Y | Y | N | Y | DK | DK | N | Y | Y | N | N | Y | DK | N | Y | Y | Y | Y | N | Y |
| Khowaja, Soomro et al. 2011 | Y | Y | Y | Y | Y | Y | N | Y | N | N | N | Y | N | N | Y | DK | Y | Y | N | Y |
| Mohamed, Shwaib et al. 2017 | Y | Y | Y | Y | Y | Y | Y | Y | Y | Y | Y | Y | N | Y | Y | Y | Y | Y | N | Y |
| Ogoina, Oyeyemi et al. 2016 | Y | Y | DK | Y | Y | DK | N | Y | DK | Y | N | N | Y | N | Y | Y | Y | Y | N | Y |
| Olowookere, Abioye-Kuteyi et al. 2015 | Y | Y | Y | Y | Y | DK | Y | Y | Y | Y | Y | Y | N | N | Y | Y | Y | Y | N | Y |
| Ozioko, Okoye et al. 2018 | Y | Y | Y | Y | Y | Y | N | Y | N | Y | N | Y | N |  | Y | Y | Y | Y | N | Y |
| Philavong, Pruvot et al. 2020 | Y | Y | Y | Y | Y | Y | Y | Y | Y | Y | Y | Y | N | Y | Y | Y | Y | Y | N | Y |
| Rizwan, Sadiq et al. 2020 | Y | Y | Y | Y | Y | DK | N | Y | N | Y | Y | Y | N | N | Y | Y | Y | Y | DK | Y |
| Schaetti, Sundaram et al. 2013 | Y | Y | N | Y | DK | DK | N | Y | DK | N | Y | Y | DK | N | Y | Y | Y | Y | N | Y |
| Sengeh, Jalloh et al. 2020 | Y | Y | Y | Y | Y | Y | N | Y | Y | Y | Y | Y | N | N | Y | Y | Y | Y | DK | Y |
| Shabani, Ezekiel et al. 2015 | Y | Y | Y | Y | Y | Y | N | Y | Y | Y | Y | Y | N | N | Y | Y | Y | Y | N | Y |
| Shakeel, Rehman et al. 2020 | Y | Y | Y | Y | Y | DK | N | Y | N | Y | Y | Y | N | N | Y | Y | Y | N | N | Y |
| Tadesse, Gebrewahd et al. 2020 | Y | Y | Y | Y | Y | Y | N | Y | Y | N | N | Y | N | N | N | N | Y | N | N | Y |
| ul Haq, Shahbaz et al. 2020 | Y | Y | Y | Y | DK | DK | N | Y | N | Y | Y | Y | DK | N | Y | Y | Y | Y | N | Y |
| Usifoh, Odigie et al. 2019 | Y | Y | Y | Y | Y | Y | N | Y | Y | N | N | Y | N | N | Y | Y | Y | N | N | Y |
| Usuwa, Akpa et al. 2020 | Y | Y | Y | Y | Y | Y | N | Y | Y | Y | Y | Y | N | N | Y | Y | Y | Y | N | Y |
| Winters, Jalloh et al. 2020 | Y | Y | Y | Y | Y | Y | N | Y | Y | Y | Y | Y | N | N | Y | Y | Y | Y | N | Y |

Y: Yes; N: No; DK: Don’t know

**RATS guidelines for qualitative research review – n=5**

| **Author(s) and year** | **Relevance of study question** | | **Appropriateness of qualitative method** | **Transparency of procedures** | | | | | | | | **Soundness of interpretive approach** | | | | | | | **Red flags** |
| --- | --- | --- | --- | --- | --- | --- | --- | --- | --- | --- | --- | --- | --- | --- | --- | --- | --- | --- | --- |
|  | **Research question explicitly stated** | **Research question justified and linked to the existing knowledge base** | **Study design described and justified e.g., why was a particular method (i.e., interviews) chosen?** | **Criteria for selecting the study sample justified and explained** | **Details of how recruitment was conducted and by whom** | **Details of who chose not to participate and why** | **Do the researchers occupy dual roles (clinician and researcher)?** | **Are the ethics of this discussed?** | **Informed consent process explicitly and clearly detailed** | **Anonymity and confidentiality discussed** | **Ethics approval cited** | **Analytic approach described in depth and justified** | **Indicators of quality** | **Findings presented with reference to existing theoretical and empirical literature** | **Strengths and limitations explicitly described and discussed** | **Evidence of following guidelines (format, word count)** | **Detail of methods or additional quotes contained in appendix** | **Written for a health sciences audience** | **Jargon; Overinterpretation; Seems anecdotal, self-evident; Consent process thinly discussed; Doctor-researcher** |
| Ayegbusi, Jegede et al. 2016 | Y | Y | N | Y | N | N | N | N | N | N | N | N | N | Y | N | Y | N | Y | N |
| Bell, Munro-Kramer et al. 2017 | Y | Y | Y | Y | Y | N | N | Y | Y | N | Y | Y | Y | N | Y | Y | N | Y | N |
| Blum, Dentz et al. 2014 | Y | Y | Y | Y | Y | N | DK | Y | Y | N | Y | Y | Y | Y | N | Y | N | Y | N |
| Englert, Kiwanuka et al. 2019 | Y | Y | N | Y | Y | N | N | Y | N | N | Y | Y | Y | Y | Y | Y | N | Y | N |
| Murele, Vaz et al. 2014 | Y | Y | Y | Y | Y | N | DK | N | N | N | N | Y | Y | Y | Y | Y | N | Y | Y |

Y: Yes; N: No; DK: Don’t know

**Mixed Methods Appraisal Tool (MMAT) - (n=6)**

| **Author(s) and year** | **Screening** | | **Qualitative** | | | | | **Quantitative nonrandomized** | | | | | **Quantitative descriptive** | | | | | **Mixed methods** | | | | |
| --- | --- | --- | --- | --- | --- | --- | --- | --- | --- | --- | --- | --- | --- | --- | --- | --- | --- | --- | --- | --- | --- | --- |
|  | Are there clear research questions? | Do the collected data allow to address the research questions? | Is the qualitative approach appropriate to answer the research question? | Are the qualitative data collection methods adequate to address the research question? | Are the findings adequately derived from the data? | Is the interpretation of results sufficiently substantiated by data? | Is there coherence between qualitative data sources, collection, analysis and interpretation? | Are the participants representative of the target population? | Are measurements appropriate regarding both the outcome and intervention (or exposure)? | Are there complete outcome data? | Are the confounders accounted for in the design and analysis? | During the study period, is the intervention administered (or exposure occurred) as intended | Is the sampling strategy relevant to address the research question? | Is the sample representative of the target population? | Are the measurements appropriate? | Is the risk of nonresponse bias low? | Is the statistical analysis appropriate to answer the research question? | Is there an adequate rationale for using a mixed methods design to address the research question? | Are the different components of the study effectively integrated to answer the research question? | Are the outputs of the integration of qualitative and quantitative components adequately interpreted? | Are divergences and inconsistencies between quantitative and qualitative results adequately addressed? | Do the different components of the study adhere to the quality criteria of each tradition of the methods involved? |
| Claude, Underschultz et al. 2019 | Y | Y | Y | Y | Y | Y | Y | DK | Y | Y | Y | Y | Y | Y | Y | DK | Y | Y | Y | Y | N | Y |
| Kamara, Mokuwa et al. 2020 | Y | Y | Y | Y | Y | Y | Y | Y | Y | Y | Y | Y | Y | Y | Y | Y | Y | Y | Y | Y | N | Y |
| Kasereka and Hawkes 2019 | Y | Y | Y | Y | Y | Y | Y | DK | Y | N | DK | Y | N | DK | Y | DK | N | Y | Y | Y | N | Y |
| Schmidt-Hellerau, Winters et al. 2020 | Y | Y | Y | Y | Y | Y | Y | Y | Y | Y | Y | Y | Y | Y | Y | Y | Y | Y | Y | Y | N | Y |
| Xu, Liu et al. 2019 | Y | Y | Y | Y | Y | Y | Y | DK | Y | Y | N | Y | DK | DK | Y | Y | Y | Y | Y | Y | N | Y |
| Xu, Liu et al. 2020 | Y | Y | Y | Y | Y | Y | Y | DK | Y | Y | N | Y | DK | DK | Y | Y | Y | Y | Y | Y | N | Y |

Y: Yes; N: No; DK: Don’t know
